# Prognostic impact of elevated lactate levels on mortality in critically ill patients with and without preadmission metformin treatment: a Danish registry-based cohort study

**DOI:** 10.1186/s13613-020-00652-0

**Published:** 2020-03-26

**Authors:** Rene A. Posma, Trine Frøslev, Bente Jespersen, Iwan C. C. van der Horst, Daan J. Touw, Reimar W. Thomsen, Maarten W. Nijsten, Christian F. Christiansen

**Affiliations:** 1 Department of Critical Care, University of Groningen, University Medical Center Groningen, Hanzeplein 1, P.O. Box 30.001, 9700 RB Groningen, The Netherlands; 2 Department of Clinical Epidemiology, Aarhus University Hospital, Aarhus, Denmark; 3 Department of Renal Medicine, Aarhus University Hospital, Aarhus, Denmark; 4 Department of Intensive Care, Maastricht University Medical Center+, Maastricht University, Maastricht, The Netherlands; 5 Department of Clinical Pharmacy and Pharmacology, University of Groningen, University Medical Center Groningen, Groningen, The Netherlands

**Keywords:** Metformin, Lactate, Effect modification, Metabolism, Diabetes mellitus

## Abstract

**Background:**

Lactate is a robust prognostic marker for the outcome of critically ill patients. Several small studies reported that metformin users have higher lactate levels at ICU admission without a concomitant increase in mortality. However, this has not been investigated in a larger cohort. We aimed to determine whether the association between lactate levels around ICU admission and mortality is different in metformin users compared to metformin nonusers.

**Methods:**

This cohort study included patients admitted to ICUs in northern Denmark between January 2010 and August 2017 with any circulating lactate measured around ICU admission, which was defined as 12 h before until 6 h after admission. The association between the mean of the lactate levels measured during this period and 30-day mortality was determined for metformin users and nonusers by modelling restricted cubic splines obtained from a Cox regression model.

**Results:**

Of 37,293 included patients, 3183 (9%) used metformin. The median (interquartile range) lactate level was 1.8 (1.2–3.2) in metformin users and 1.6 (1.0–2.7) mmol/L in metformin nonusers. Lactate levels were strongly associated with mortality for both metformin users and nonusers. However, the association of lactate with mortality was different for metformin users, with a lower mortality rate in metformin users than in nonusers when admitted with similar lactate levels. This was observed over the whole range of lactate levels, and consequently, the relation of lactate with mortality was shifted rightwards for metformin users.

**Conclusion:**

In this large observational cohort of critically ill patients, early lactate levels were strongly associated with mortality. Irrespective of the degree of hyperlactataemia, similar lactate levels were associated with a lower mortality rate in metformin users compared with metformin nonusers. Therefore, lactate levels around ICU admission should be interpreted according to metformin use.

## Background

Lactate is the most robust early routine laboratory marker for outcome in critically ill patients [1, 2]. Lactate levels are generally elevated as part of the stress response or due to impaired lactate utilisation [3]. Therefore, lactate is increasingly measured to stratify risk and to monitor the course of critical illness [3].

Metformin is the recommended first-line glucose-lowering medication for the management of type 2 diabetes mellitus [4]. Although its mechanism of action remains debated [5], metformin has been shown to selectively reduce hepatic lactate uptake and subsequent glucose production through mild inhibition of mitochondria [6–9]. Conversion of lactate into glucose in the liver, as part of the Cori cycle, plays a pivotal role in lactate metabolism during physiological stress [3, 10]. Inhibition of this cycle by metformin may result in more marked hyperlactataemia in times of critical illness [11]. On the other hand, some patients with renal dysfunction accumulate metformin and develop severe lactic acidosis, which is caused by excessive systemic and hepatic mitochondrial inhibition [11, 12].

Given its mechanism of action, metformin may contribute to plasma lactate elevation without the patient being more severely ill than individuals with similar lactate levels [13]. Compared to critically ill patients not using metformin, some studies reported that metformin users have higher lactate levels at admission without a concomitant increase in mortality [14–17], although others could not corroborate these findings [18–22]. To determine whether the association between early lactate level and mortality during critical illness is different in metformin users compared with metformin nonusers, we studied a large cohort of patients admitted to intensive care units (ICU) in northern Denmark.

## Methods

### Setting and inclusion

Data were collected through the unambiguous individual-level linkage between population-based medical registries and databases using the unique central personal identification number assigned to each Danish resident at birth or upon immigration [23]. We included subjects aged 18 years and older who, between January 1st, 2010 and July 31st, 2017, had a hospitalisation in northern Denmark (i.e., the Northern and Central Denmark Regions, Additional file 1: Appendix S1) that included admission to the ICU. Patients were subsequently identified from the previously validated Danish Intensive Care Database (DID) [24], which covers virtually all patients admitted to an ICU in Denmark [25]. During the study period, all 10 hospitals in northern Denmark were connected to the population-based regional laboratory research database, which covers both in- and outpatient measurements [26]. To ensure the availability of a complete history of laboratory data, we required that the included patients lived in the area for at least 1 year [27]. Of patients fulfilling the criteria above, we then included the first ICU admission of patients with at least one reported blood lactate level between 12 h before until 6 h after ICU admission. This time frame was chosen to capture lactate levels that are typically taken into consideration to assess disease severity and monitor initiated treatment [28, 29]. Of patients without a lactate measurement, we recorded data regarding the first ICU admission within the inclusion period. To avoid immortal time bias, we excluded patients who died within 6 h after ICU admission [30].

### Preadmission metformin use

Prescription data were obtained from the nationwide Danish National Health Service Prescription Database [31]. For each patient, we identified all filled prescriptions for antihyperglycemic drugs (Additional file 1: Appendix S2) [27, 31]. Metformin users were defined as patients with at least one prescription for metformin as monotherapy or in combination with any other antihyperglycemic drug within 90 days before ICU admission. This period was chosen because prescriptions rarely are issued for more than 3 months [27].

### Exposure and outcome

The exposure was the mean of all lactate measurements obtained 12 h before until 6 h after ICU admission (Additional file 1: Fig. S1). The outcome was death within 30 days following ICU admission. Data for all Danish inhabitants regarding vital status, emigration, and residency were obtained from the Danish Civil Registration System (DCRS) [23]. Patients were censored at the date of emigration or at the end of follow-up, whichever came first. We investigated whether metformin use interacted with the relation between lactate and mortality (i.e., the effect measure modification) [32].

### Patient characteristics

We used the DCRS to obtain data on age and sex. Comorbidity level, according to the Charlson Comorbidity Index, was estimated using inpatient and outpatient hospital contacts recorded in the Danish National Patient Registry (DNPR) within 10 years before ICU admission [33]. The primary diagnosis of hospitalisation in which the index ICU admission occurred, and the type of admission was obtained similarly from the DNPR [34]. Treatment initiated during ICU admission and the Simplified Acute Physiology Score (SAPS) II score was retrieved from the DID [25, 35]. We identified diabetes mellitus using an algorithm incorporating any previous inpatient or outpatient clinical diagnosis of diabetes 10 years before ICU admission, any prescription for an oral antihyperglycemic drug or insulin 90 days before ICU admission, or a glycosylated haemoglobin A1c (HbA1c) level ≥ 6.5% (48 mmol/mol) within a year before admission [27, 36]. Preadmission renal function was determined by calculating the mean plasma creatinine concentration 365 days until 7 days before ICU admission [37]. The estimated glomerular filtration rate (eGFR) was calculated using the Chronic Kidney Disease Epidemiology Collaboration equation assuming Caucasian race [38, 39]. Data on chronic renal replacement therapy (RRT) use the year before ICU admission were obtained from the DNPR [34].

### Statistical analyses

All missing variables, provided as footnotes in Table 1, were imputed via multivariate imputation by chained equations with 100 imputation sets, using all data presented in Table 1 and Additional file 1: Table S2 as predictors to match. Estimates were pooled according to Rubin’s rules [40]. Differences with 95% confidence interval (CI) in median lactate level between metformin nonusers and users were computed by bootstrapping the median difference with 1000 resamples [41]. Additionally, we compared the proportion of metformin users and nonusers with a maximum lactate of ≥ 2 mmol/L, which is often considered as the threshold level identifying severe critical illness, such as septic shock [42]. Thirty-day mortality was computed as one minus the Kaplan–Meier survival estimate.

**Table 1:** Patient characteristics stratified by lactate level category

Characteristic	Total	< 1.4	1.4 to 2.0	2.0 to 3.2	3.2 to 5.0	5.0 to 10.0	≥ 10
No. of patients	37,293	14,572	8705	6831	3694	2544	947
Age, years	68 [56–77]	68 [57–77]	67 [55–77]	67 [53–77]	68 [55–77]	68 [56–77]	66 [55–74]
Male	21,244 (57)	8010 (55)	4971 (57)	3999 (59)	2125 (58)	1552 (61)	587 (62)
Charlson Comorbidity Index							
0	13,769 (37)	5122 (35)	3311 (38)	2667 (39)	1337 (36)	954 (38)	378 (40)
1–2	14,005 (38)	5696 (39)	3248 (37)	2468 (36)	1344 (36)	931 (37)	318 (34)
≥ 3	9519 (26)	3754 (26)	2146 (25)	1696 (25)	1013 (27)	659 (26)	251 (27)
Preadmission disease							
Diabetes mellitus	7703 (21)	2790 (19)	1787 (21)	1404 (21)	812 (22)	673 (26)	237 (25)
Myocardial infarction	2630 (7)	1108 (8)	621 (7)	425 (6)	251 (7)	162 (6)	63 (7)
Congestive heart failure	3520 (9)	1424 (10)	837 (10)	594 (9)	341 (9)	236 (9)	88 (9)
Peripheral vascular disease	4104 (11)	1648 (11)	998 (11)	681 (10)	400 (11)	278 (11)	99 (10)
Cerebrovascular disease	5065 (14)	2084 (14)	1138 (13)	876 (13)	511 (14)	333 (13)	123 (13)
Chronic pulmonary disease	6863 (18)	3011 (21)	1563 (18)	1151 (17)	642 (17)	387 (15)	109 (12)
Cancer	6735 (18)	2771 (19)	1525 (18)	1238 (18)	683 (18)	401 (16)	117 (12)
Mild-to-severe liver disease	1531 (4)	384 (3)	315 (4)	292 (4)	218 (6)	217 (9)	105 (11)
Renal function during 1 year before ICU admission^a^							
eGFR, mL/min/1.73 m^2^	80 [59–95]	80 [58–95]	81 [61–95]	81 [59–96]	80 [59–96]	81 [59–96]	85 [62–99]
≥ 60 mL/min/1.73 m^2^	22,770 (74)	9029 (73)	5399 (76)	4041 (75)	2234 (74)	1506 (74)	561 (77)
45–60 mL/min/1.73 m^2^	3773 (12)	1531 (12)	808 (11)	713 (13)	371 (12)	263 (13)	87 (12)
30–45 mL/min/1.73 m^2^	2402 (8)	1020 (8)	528 (7)	398 (7)	242 (8)	162 (8)	52 (7)
< 30 mL/min/1.73 m^2^	1641 (5)	755 (6)	352 (5)	244 (5)	163 (5)	98 (5)	29 (4)
Dialysis before ICU admission^b^	344 (1)	139 (1)	85 (1)	52 (1)	43 (1)	19 (1)	6 (1)
Antihyperglycemic therapy							
Metformin	3183 (9)	1047 (7)	758 (9)	596 (9)	353 (10)	300 (12)	129 (14)
Sulfonylureas	899 (2)	309 (2)	233 (3)	150 (2)	104 (3)	72 (3)	31 (3)
Insulin	2343 (6)	853 (6)	551 (6)	428 (6)	236 (6)	220 (9)	55 (6)
Other antihyperglycemic agents	691 (2)	206 (1)	158 (2)	131 (2)	88 (2)	72 (3)	36 (4)
HbA1c within 4 weeks before ICU admission, %^c^	5.9 [5.5–6.7]	5.9 [5.5–6.5]	6.0 [5.5–6.7]	6.1 [5.5–7.0]	6.1 [5.5–6.9]	6.1 [5.5–7.0]	5.9 [5.5–7.0]
ICU admission type^d^							
Medical	17,926 (52)	5864 (43)	4033 (50)	3575 (56)	2188 (63)	1622 (68)	644 (73)
Emergency surgery	10,683 (31)	4111 (30)	2506 (31)	2041 (32)	1078 (31)	722 (30)	225 (26)
Elective surgery	6193 (18)	3623 (27)	1576 (19)	738 (12)	187 (5)	56 (2)	13 (1)
SAPS-II score^e^	40 [30–52]	35 [26–45]	38 [29–49]	41 [31–53]	47 [36–60]	54 [42–67]	62 [50–76]
Primary diagnostic category							
Cardiovascular	8466 (23)	3668 (25)	1931 (22)	1183 (17)	721 (20)	667 (26)	296 (31)
Respiratory, incl. pneumonia	5244 (14)	2223 (15)	1251 (14)	972 (14)	498 (13)	248 (10)	52 (5)
Infection or sepsis, excl. pneumonia	3425 (9)	1054 (7)	789 (9)	689 (10)	476 (13)	335 (13)	82 (9)
Gastrointestinal and liver disease	4215 (11)	1635 (11)	923 (11)	783 (11)	435 (12)	313 (12)	126 (13)
Neoplasms	3899 (10)	1777 (12)	971 (11)	713 (10)	282 (8)	125 (5)	31 (3)
Trauma and poisoning	5014 (13)	1887 (13)	1247 (14)	1109 (16)	446 (12)	255 (10)	70 (7)
Endocrinology	1013 (3)	381 (3)	203 (2)	226 (3)	93 (3)	79 (3)	31 (3)
Other	6017 (16)	1947 (13)	1390 (16)	1156 (17)	743 (20)	522 (21)	259 (27)
Laboratory values 12 h before until 6 h after ICU admission							
First lactate, mmol/L	1.6 [1.0–3.0]	0.9 [0.7–1.2]	1.7 [1.4–2.0]	2.6 [2.1–3.3]	4.2 [3.4–5.5]	7.4 [5.6–9.6]	14.0 [11.4–17.0]
Mean lactate, mmol/L	1.6 [1.1–2.8]	0.9 [0.8–1.1]	1.6 [1.5–1.8]	2.5 [2.3–2.8]	3.9 [3.5–4.4]	6.6 [5.7–7.9]	13.0 [11.3–15.8]
Maximum lactate, mmol/L	2.1 [1.3–3.7]	1.1 [0.9–1.4]	2.0 [1.8–2.5]	3.2 [2.7–4.0]	5.1 [4.3–6.4]	8.8 [7.1–10.8]	15.3 [13.0–18.9]
No. of lactate measurements	4 [2–6]	3 [2–5]	4 [2–6]	4 [2–6]	4 [2–6]	4 [2–7]	3 [1–7]
Mean glucose, mmol/L^f^	8.1 [6.7–10.1]	7.3 [6.3–8.6]	8.3 [7.0–9.9]	8.8 [7.1–10.9]	9.4 [7.4–12.1]	10.0 [7.4–13.6]	9.8 [6.8–14.1]
ICU treatment							
Mechanical ventilation	15,594 (42)	5377 (37)	3441 (40)	2839 (42)	1838 (50)	1466 (58)	633 (67)
Inotropes or vasopressors	13,081 (35)	3712 (25)	2854 (33)	2641 (39)	1811 (49)	1461 (57)	602 (64)
Renal replacement therapy	1919 (5)	440 (3)	316 (4)	351 (5)	291 (8)	331 (13)	190 (20)
ICU length of stay, days^g^	1.1 [0.7–2.8]	1.0 [0.7–2.1]	1.0 [0.7––2.8]	1.2 [0.6–3.3]	1.5 [0.7–4.0]	1.5 [0.6–4.3]	1.0 [0.4–3.3]
Hospital length of stay, days	10 [5–20]	10 [6–20]	11 [6–21]	10 [5–21]	10 [4–21]	9 [3–21]	5 [1–15]

Blood lactate level category based on quintiles and clinically relevant boundaries in mmol/L. Data are expressed as n (%) or median [IQR]

^a^Data missing for 6707 (18%) patients

^b^As per Danish regulation, frequencies smaller than five patients not being zero are not reported

^c^Data missing for 31,373 (84%) patients

^d^Data missing for 2491 (7%) patients

^e^Data missing for 25,932 (70%) patients

^f^Data missing for 807 (2%) patients

^g^Data missing for 2885 (8%) patients

To assess the association of lactate with outcome, we computed hazard ratios (HR) with 95% CI for death using multivariable Cox proportional hazards regression analysis. This model was chosen because the hazard ratio approximates the rate ratio at the expected event rate in our study, making this estimator easier to interpret than, for example, odds ratios [43]. The proportional hazards assumption was checked graphically and found to be met. The model was adjusted for age, sex, preadmission plasma creatinine concentration, HbA1c level, and several preadmission comorbidities (Additional file 1: Appendix S3). Creatinine was included since decreased renal function may, directly and indirectly, lead to impaired lactate metabolism [44].

The association of lactate level as a continuous variable with mortality for metformin nonusers and users was modelled using restricted cubic splines with four equally spaced knots, of which the location was based on quantiles. Subsequently, we categorised the lactate level, based on the second, third, and fourth quintiles (1.4, 2.0, and 3.2 mmol/L, respectively) and two custom-defined boundaries (5.0 and 10.0 mmol/L, respectively), into six groups. Lactate groups, stratified by metformin use, were entered into the model as a categorical variable. To quantify interaction on an additive scale, we calculated the relative excess risk due to interaction (RERI), of which the 95% CI was based on the delta method [32].

To investigate whether potential bias was introduced by imputation, we also performed all analyses in a dataset restricted to complete cases [40]. As HbA1c was only reported in 15% of the patients, the complete case analysis was performed without including HbA1c as a covariable. We also performed our primary analysis in subgroups, including (1) patients with diabetes, (2) patients admitted for elective surgery, and (3) after stratification by preadmission renal function. The last subgroup was chosen because metformin-associated lactic acidosis might be more prevalent among metformin users with chronic kidney disease [11, 45, 46]. Additionally, we corrected for potential surveillance bias by adjusting for the number of lactate measurements in a separate analysis. Details regarding covariates used in the models for each subgroup are outlined in Additional file 1: Appendix S4. To further confirm the robustness of our findings, we performed the primary analysis using the first and the maximum lactate concentration as exposure. We also applied a logistic regression model to determine the association of lactate level with mortality.

Data were analysed with R version 3.5.1 (R Foundation for Statistical Computing, Vienna, Austria), and the *rms* package was used to model restricted cubic splines. The Danish Data Protection Agency approved the study (record number 2015-57-0002, Aarhus University record number 2016-051-000001/432). According to Danish law, no ethical approval or informed consent was required for this registry-based study.

## Results

### Inclusion and patient characteristics

**Fig. 1:**
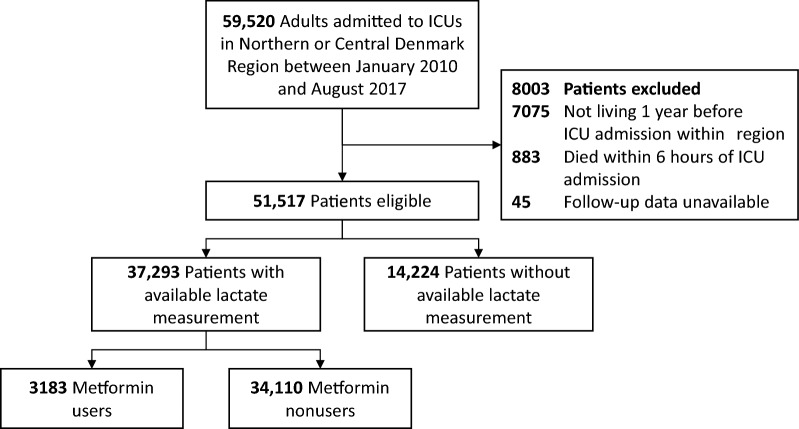
Flowchart of included patients

Of 59,520 adult ICU patients admitted during the study period, 51,517 patients were considered eligible for this study (Fig. 1). In total, 37,293 (72%) patients had at least one lactate level 12 h before until 6 h after ICU admission and were included in the final analysis (Additional file 1: Table S1). Lactate levels were more often not available at the beginning of data capture (e.g., in 2010 or 2011) and in patients with a relatively lower risk profile. Lactate levels were more often reported for metformin users than nonusers (80% vs. 72%). Three patients were lost to follow-up within 30 days after inclusion.

Median (interquartile range, IQR) age was 68 (56–77) years, 57% were male, and 3183 (9%) patients used metformin. Four metformin users did not have a diagnosis of diabetes mellitus, but these patients had a diagnosis of polycystic ovarian syndrome recorded within 10 years before ICU admission. The proportion of patients with diabetes increased in patients admitted with higher lactate levels (Table 1). The percentage of metformin users also increased in patients admitted with higher lactate levels, whereas this trend was not observed for insulin, sulfonylureas, or other glucose-lowering drugs. Patients admitted with high lactate levels more often had a history of mild-to-severe liver disease. Compared with nonusers, metformin users more often had a history of chronic pulmonary and cardiovascular disease (Additional file 1: Table S3). The median (IQR) preadmission eGFR was 80 (59–95) mL/min/1.73 m^2^, which was similar for patients when categorised according to either lactate level or metformin use. However, a larger proportion of nonusers had severe chronic kidney disease and required dialysis before ICU admission. The SAPS-II score increased when admitted with higher lactate levels, as was the number of patients requiring mechanical ventilation, inotropes or vasopressors, and RRT. While the SAPS-II score was similar for metformin users and nonusers, metformin users more often were treated with mechanical ventilation, inotropes or vasopressors, and RRT.

### Lactate level and outcome

In 12 h before until 6 h after ICU admission, lactate was measured a median (IQR) 4 (2–6) times per patient. The number of measurements was not different for patients admitted with increased lactate levels and did not differ between metformin users and nonusers (Table 1 and Additional file 1: Table S3). The median (IQR) lactate level was 1.8 (1.2–3.2) mmol/L for metformin users and 1.6 (1.0–2.7) mmol/L for metformin nonusers, resulting in a mean (95% CI) difference of 0.2 (0.2 to 0.3) mmol/L (Additional file 1: Fig. S2, S3). Compared with nonusers, metformin users more often had a maximum lactate level ≥ 2 mmol/L (43% vs. 38%, Additional file 1: Fig. S3). In total, 7768 (21%) patients died during 30 days of follow-up.

**Fig. 2:**
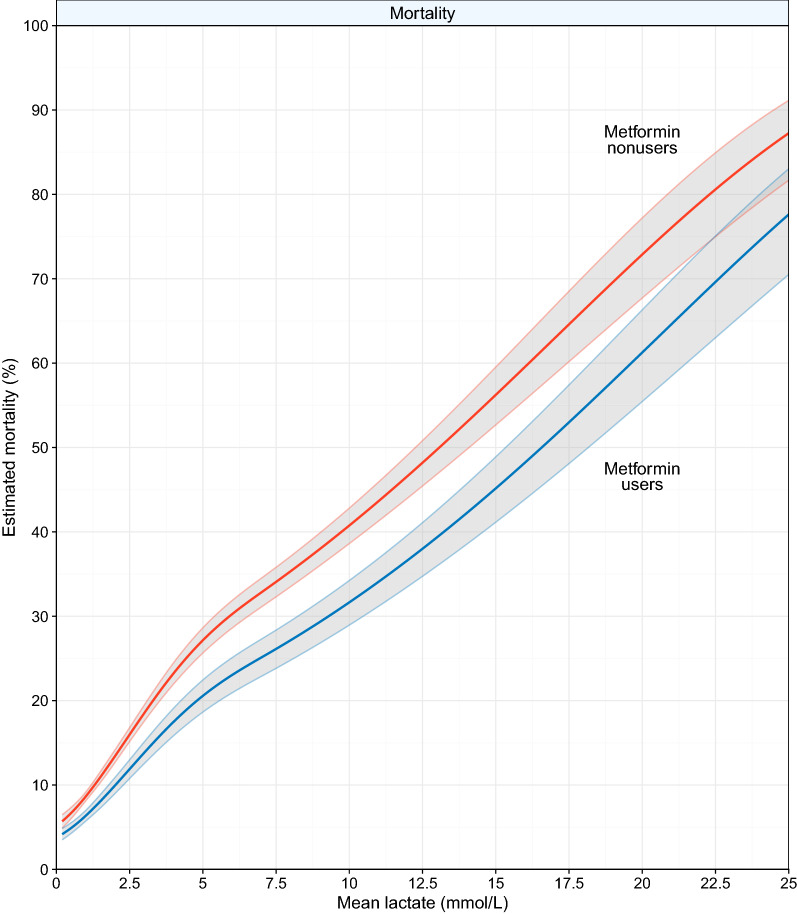
Association of lactate level with estimated 30-day mortality for metformin users and nonusers. Data were computed using the one minus the Kaplan–Meier survival estimate. Restricted cubic splines were constructed with four evenly spaced knots based on quantiles. A multiply imputed dataset was used for this analysis. The grey area represents the 95% confidence interval

**Fig. 3:**
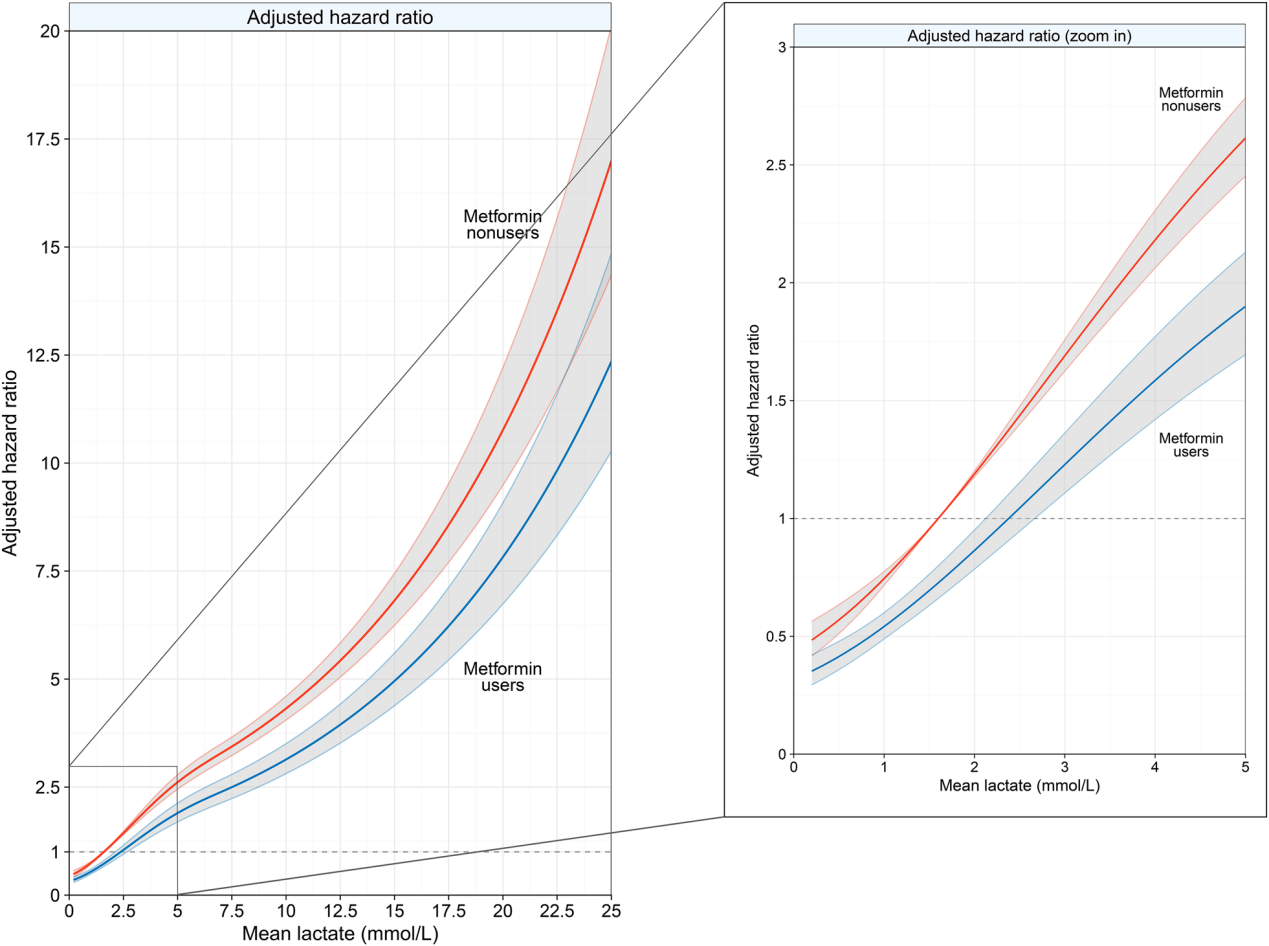
Association of lactate level with adjusted hazard ratio for 30-day mortality among metformin users and nonusers in the total cohort. The panel on the right is a zoomed-in version of the left panel and corresponds with the range of lactate levels that includes the largest proportion of patients. Data were fit by a multivariable-adjusted Cox regression model based on restricted cubic splines constructed with four evenly spaced knots. A multiply imputed dataset was used for this analysis. The grey area represents the 95% confidence interval

Lactate levels were strongly associated with mortality (Fig. 2). Over the whole range of lactate levels, metformin users had a lower mortality rate than metformin nonusers. Therefore, the relation of lactate with mortality was shifted rightwards for metformin users (Figs. 2, 3). Compared with metformin users with a lactate level < 1.4 mmol/L, nonusers with a lactate < 1.4 mmol/L had essentially the same mortality risk (Table 2). Compared to metformin nonusers with a lactate < 1.4 mmol/L, metformin nonusers with a lactate between 1.4 and 2.0 mmol/L had a higher mortality risk (HR 1.45, 95% CI 1.35–1.55), while this was less pronounced in metformin users with a lactate between 1.4 and 2.0 mmol/L (HR 1.15, 95% CI 0.94–1.39). The risk further increased in patients admitted with higher lactate categories for both metformin users and nonusers. However, this increase in risk was less pronounced for metformin users than nonusers, which was confirmed by interaction of which the magnitude increased for patients presenting with higher lactate levels (Table 2).

**Table 2:** Thirty-day mortality, hazard ratios, and the relative excess risk due to interaction by lactate category for metformin nonusers and users

Lactate (mmol/L)	Events/no. at risk (%)		Crude HR (95% CI)		Adjusted HR (95% CI)^a^		RERI (95% CI)^b^
	Metformin nonusers	Metformin users	Metformin nonusers	Metformin users	Metformin nonusers	Metformin users	
< 1.4	1784/13,525 (13%)	136/1047 (13%)	Reference	0.98 (0.82 to 1.16)	Reference	0.95 (0.80 to 1.14)	
1.4 to 2.0	1400/7947 (18%)	114/758 (15%)	1.37 (1.28 to 1.47)	1.15 (0.95 to 1.39)	1.45 (1.35 to 1.55)	1.15 (0.94 to 1.39)	− 0.25 (− 0.54 to 0.03)
2.0 to 3.2	1422/6235 (23%)	116/596 (19%)	1.84 (1.72 to 1.97)	1.53 (1.27 to 1.85)	2.00 (1.87 to 2.15)	1.53 (1.26 to 1.86)	− 0.42 (− 0.77 to − 0.07)
3.2 to 5.0	1082/3341 (32%)	84/353 (24%)	2.80 (2.60 to 3.02)	1.90 (1.53 to 2.36)	2.99 (2.77 to 3.22)	1.86 (1.49 to 2.33)	− 1.08 (− 1.55 to − 0.60)
5.0 to 10.0	964/2244 (43%)	105/300 (35%)	4.17 (3.86 to 4.51)	3.16 (2.59 to 3.84)	4.58 (4.24 to 4.96)	3.10 (2.54 to 3.80)	− 1.43 (− 2.13 to − 0.74)
≥ 10.0	498/818 (61%)	63/129 (49%)	7.60 (6.88 to 8.39)	5.20 (4.04 to 6.69)	9.24 (8.35 to 10.21)	5.51 (4.27 to 7.12)	− 3.68 (− 5.30 to − 2.05)

Hazard ratio (HR) with 95% confidence interval (CI) was computed using Cox proportional regression analysis based on a multiply imputed datasets

^a^Adjusted for age, gender, last HbA1c measurement within 4 weeks before ICU admission, mean of all creatinine measurements 1 year before ICU admission, and previous diagnosis of myocardial infarction, congestive heart failure, peripheral artery disease, cerebrovascular disease, dementia, connective tissue disease, peptic ulcer disease, chronic pulmonary disease, mild-to-severe liver disease, any tumour, metastatic solid tumour, leukaemia, and lymphoma, respectively, 10 years before ICU admission. Codes used for each variable are stated in Additional file 1: Appendix S1

^b^Relative excess risk due to interaction (RERI) quantifies interaction on an additive scale, which approaches zero in the absence of interaction

### Sensitivity analyses

Our observations did not alter materially when we: (1) restricted our analysis to complete cases; (2) adjusted for the number of lactate measurements; (3) applied a logistic regression model to determine the relation of lactate with outcome, or (4) used first or maximum lactate level as exposure (Additional file 1: Figs. S4–S6 and Tables S4–S6). Although metformin users had baseline characteristics and mortality rate different from patients with diabetes not using metformin (Additional file 1: Tables S7, S8 and Fig. S2), the relation of lactate with mortality was shifted rightwards for metformin users in this subgroup as well (Additional file 1: Table S9, Fig. S7). Among 6193 patients undergoing elective surgery, 672 (11%) patients used metformin (Additional file 1: Table S10). Lactate levels were also associated with mortality in this small subgroup, but we did not observe a clear effect modification by metformin use (Additional file 1: Table S11 and Fig. S8). When stratified by preadmission renal function (Additional file 1: Table S12–S14, Fig. S2), effect modification by metformin use was more pronounced in patients with an eGFR smaller than 60 mL/min/1.73 m^2^ when categorising lactate levels (Additional file 1: Table S15), but we could not fully substantiate this finding when modelling lactate as a continuous variable (Additional file 1: Fig. S9).

## Discussion

In this registry-based cohort study of more than 37,000 patients admitted to ICUs in northern Denmark, we confirmed a strong association of early lactate elevation with adverse outcomes. Over the whole range of lactate levels, metformin users had lower mortality than metformin nonusers. The association of lactate with mortality was thus shifted rightwards for metformin users, meaning that metformin users have a lower mortality rate when admitted with similar lactate levels as metformin nonusers. Therefore, the prognostic value of lactate was modified by metformin use in our study.

The most straightforward explanation of our results is that lactate metabolism is affected by metformin due to its prime mode of action, inhibition of gluconeogenesis (i.e., synthesis of glucose from circulating lactate) [5, 7, 11]. A related explanation could be that metformin users are more susceptible to generate lactate because of systemic but mild suppression of mitochondria [8, 9, 13], although this has hitherto only been demonstrated in cases with toxic metformin levels [12]. Metformin users generally have more comorbidities than the total population, which might affect the decision to admit these patients at an earlier stage of critical illness to the ICU. For that reason, selection bias might contribute to the fact that metformin users have a more favourable outcome. However, the same rightward shift of the association of lactate with mortality was observed when restricting to patients with diabetes, among whom metformin users had a lower risk profile compared to metformin nonusers with diabetes. Likewise, metformin users may carry a lower mortality risk compared with nonusers when admitted to an ICU, as observed previously in a Danish nationwide registry-based study and by other observational studies [27, 47, 48]. Preclinical data indicate that metformin is associated with reduced inflammation, thrombosis, and apoptosis [49]. However, randomiaed controlled trials do not provide evidence that metformin affected the mortality rate of critically ill patients, although they are underpowered for this outcome [50–52]. Larger trials are warranted to evaluate further the use of metformin as adjuvant or even protective agent in intensive care.

Importantly, by including more than 37,000 critically ill patients from 10 ICUs, our study is one of the largest studies to investigate the association of circulating lactate levels with outcome. Moreover, this is the largest study investigating the impact of metformin on this relation. Our hypothesis [13] was sparked by a previous study including 162 patients admitted to an ICU with overt hyperlactataemia and septic shock [15]. In that study, metformin users had a better prognosis compared with nonusers, but effect modification by metformin use was not assessed. Consistent with our results, others report that the optimal lactate level on the receiver operating characteristics curve was higher in critically ill metformin users than nonusers [14]. In a single-centre study including 1947 patients with suspected sepsis at the emergency department, metformin use also modified the association of lactate with outcome [16]. Other studies did not observe differences in lactate level between metformin users and nonusers [18, 19–22]. Of note, most of these studies included patients with a relatively low-risk profile, such as after cardiovascular surgery [20–22] or patients not admitted to an ICU [19].

As the presentation with high lactate levels is associated with an unfavourable outcome, an important practical clinical consequence is that metformin users do not carry the same risk as metformin nonusers when admitted with similar lactate levels. For example, the mortality rate in patients with a lactate of 5–10 mmol/L was 43% in metformin nonusers, whereas metformin users with this lactate category had a mortality rate of 35%. Therefore, clinicians could be more lenient regarding lactate as a prognostic marker for metformin users compared to nonusers with a similar lactate level. Even for lactate levels within the reference range, a mild increase in lactate was associated with a higher mortality risk for metformin nonusers. Additionally, patients with septic shock can be identified according to the Sepsis-3 definition by a lactate ≥ 2 mmol/L [42]. In our general ICU population, metformin users crossed this threshold level more often than nonusers. Although not investigated in the current study, metformin users might fulfil these criteria at an earlier stage of disease severity than nonusers with septic shock.

Metformin has been repeatedly, although infrequently, associated with lactic acidosis [11]. Currently, the incidence of metformin-associated lactic acidosis remains unclear but has been reported as 3 to 10 cases per 100,000 person-years [46]. We found that the magnitude of effect modification by metformin was more pronounced when lactate levels increased. This observation is not surprising since increasing metformin levels increasingly affect lactate metabolism, as attested in extremo in patients with metformin-associated lactic acidosis [53]. As expected from its mitochondrial inhibitory effect [5, 8, 9], both increased lactate generation and impaired lactate utilisation are explanations for the relatively higher lactate levels in metformin users than nonusers within the same mortality category. Since the major mode of metformin elimination is the excretion of unchanged drug in urine, patients with reduced kidney function are more prone to develop toxic metformin levels [11, 46]. In line with our expectations and previous studies [45, 54], effect modification by metformin was more pronounced in patients with chronic kidney disease.

The strengths of our study included its large sample size, high data validity, and practically no loss to follow-up. Furthermore, all data were prospectively collected and independent of the current study, thereby limiting the risk of information bias. The findings from the present study should be considered in light of several limitations. Lactate levels were not measured or reported in 28% of eligible patients. Although some can be explained by not all hospitals reporting lab data at the beginning of the study period, lactate levels were more often measured in metformin users compared with nonusers, which might have resulted in selection bias. Nearly all patients using metformin had diabetes, while most metformin nonusers did not, introducing potential confounding by indication [55]. However, our results were not altered when restricting our dataset to patients with diabetes exclusively. Moreover, diabetes has been associated with attenuation of lactate levels after cardiac surgery [56]. Having diabetes would, therefore, counteract any effects of metformin on lactate, thus biasing our results towards no difference and therefore not changing our conclusions. Still, our non-randomised design may have led to confounding by factors related to the choice of metformin as treatment. To address this possibility, we extensively adjusted our results for a wide range of potential confounders, which did not materially alter our results. Because the exposure in this study was a relatively short time window, static rather than dynamic indices of lactataemia were used to predict mortality. Of dynamic indices, time-weighted average lactate levels are shown to have the strongest association with both ICU and hospital mortality [57], which we approximated by calculating the mean of all lactate levels around ICU admission. Given the current inclusion criteria, we were not able to assess lactate kinetics adequately, which is a limitation of our study.

## Conclusions

In this large cohort of critically ill patients admitted to ICUs in northern Denmark, early lactate levels were strongly associated with mortality. Irrespective of the degree of hyperlactataemia, similar lactate levels were associated with a lower mortality rate in metformin users compared to metformin nonusers. Our findings suggest that metformin interacts with lactate metabolism in critically ill patients. Therefore, lactate levels around ICU admission should be interpreted according to metformin use.

## Supplementary information

**Additional file 1:** Additional figures, tables and appendices.

## Data Availability

Parts of the data that support the findings of this study are available from the Danish Health Data Authority (Sundhedsdatastyrelsen), but restrictions apply to the availability of these data, which were used under license for the present study and so are not publicly available.

## Abbreviations

ICU: Intensive care unit
DID: Danish Intensive Care Database
DCRS: Danish Civil Registration System
DNPR: Danish National Patient Registry
SAPS: Simplified Acute Physiology Score
HbA1c: Glycosylated haemoglobin A1c
eGFR: Estimated glomerular filtration rate
RRT: Renal replacement therapy
CI: Confidence interval
HR: Hazard ratio
RERI: Relative excess risk due to interaction
IQR: Interquartile range
