# Supplementary material for: Prognostic impact of elevated lactate levels on mortality in critically ill patients with and without preadmission metformin treatment: a Danish registry-based cohort study
Source: Ann Intensive Care. 2020 Mar 26;10:36. doi: 10.1186/s13613-020-00652-0 (PMC7098407; doi:10.1186/s13613-020-00652-0)
Supplement: Supplementary file 1 — Additional file 1: Additional figures, tables and appendices. [file 13613_2020_652_MOESM1_ESM.pdf]

**Prognostic impact of elevated lactate levels on mortality in critically ill patients with and without preadmission metformin treatment: a Danish registry-based cohort study**

Rene A. Posma, Trine Frøslev, Bente Jespersen, Iwan C.C. van der Horst, Daan J. Touw, Reimar W. Thomsen, Maarten W. Nijsten, and Christian F. Christiansen

This supplementary material has been provided by the authors to give readers additional information about their work.

**Additional Figures**

Figure S1 – Schematic representation of the study design. ....2
Figure S2 – Tukey's boxplot of mean lactate levels and mortality rate for patients in the total cohort, among patients with diabetes mellitus, undergoing elective surgery, or with a normal renal function or chronic kidney disease one year before ICU admission. .3
Figure S3 – Distribution of metformin users and nonusers based on mean or maximum lactate level....4
Figure S4 - Association of lactate level with estimated 30-day mortality for metformin users and nonusers when restricted to complete cases....5
Figure S5 – Association of mean lactate level with adjusted odds ratio for 30-day mortality for metformin users and nonusers in the total cohort....6
Figure S6 – Association of first and maximum lactate level, respectively, measured within 12 hours before until 6 hours after ICU admission with estimated 30-day mortality and its corresponding adjusted hazard ratio for metformin users and nonusers in the total cohort. ....7
Figure S7 – Association of lactate level with estimated 30-day mortality and its corresponding adjusted hazard ratio for metformin users and nonusers when including patients with diabetes mellitus. ....8
Figure S8 – Association of lactate level with estimated 30-day mortality and its corresponding adjusted hazard ratio for metformin users and nonusers when only including patients undergoing elective surgery. ....9
Figure S9 – Association of lactate level with estimated 30-day mortality and its corresponding adjusted hazard ratio for metformin users and nonusers among patients with a normal renal function or chronic kidney disease one year before ICU admission. ....10

**Additional Tables**

Table S1 – Characteristics of patients with and without available lactate measurements ....11
Table S2 – Individual components of the Charlson Comorbidity Index in the full cohort ....13
Table S3 – Characteristics of metformin nonusers and metformin users ....14
Table S4 – Thirty-day mortality, hazard ratios, and the relative excess risk due to interaction by lactate category for metformin nonusers and users when restricting to complete cases ....17
Table S5 – Thirty-day mortality, hazard ratios, and the relative excess risk due to interaction by lactate category for metformin nonusers and users when correcting for number of lactate measurements ....18
Table S6 – Thirty-day mortality, odds ratios, and the relative excess risk due to interaction by lactate category for metformin nonusers and users....19
Table S7 – Characteristics of patients with diabetes mellitus stratified by lactate level category ....20
Table S8 – Characteristics of metformin nonusers and users, when only including patients with diabetes....23
Table S9 – Thirty-day mortality, hazard ratios, and the relative excess risk due to interaction by lactate category for metformin nonusers and users among patients with diabetes mellitus....25
Table S10 – Characteristics of patients that underwent elective surgery stratified by metformin use and stratified by lactate level category....26
Table S11 – Thirty-day mortality, hazard ratios, and the relative excess risk due to interaction by lactate category for metformin nonusers and users undergoing elective surgery....28
Table S12 – Characteristics of metformin nonusers and metformin users, stratified by preadmission eGFR ....29
Table S13 – Characteristics of patients with preadmission eGFR ≥60 ml/min/1.72m$^2$, stratified by lactate level category....31
Table S14 – Characteristics of patients with preadmission eGFR <60 ml/min/1.72m$^2$, stratified by lactate level category ....33
Table S15 – Thirty-day mortality, hazard ratios, and the relative excess risk due to interaction by lactate category for metformin nonusers and users, when comparing two subgroups based on preadmission estimated glomerular filtration rate ....35

**Appendices**

Appendix 1 – List of hospitals included in the study....37
Appendix 2 – Codes used in this study....38
Appendix 3 – Covariates entered in the models ....41
Appendix 4 – Additional methods regarding subgroups ....42

**Additional file 1 -** Prognostic impact of elevated lactate levels on mortality in critically ill patients with and without preadmission metformin treatment: a Danish registry-based cohort study

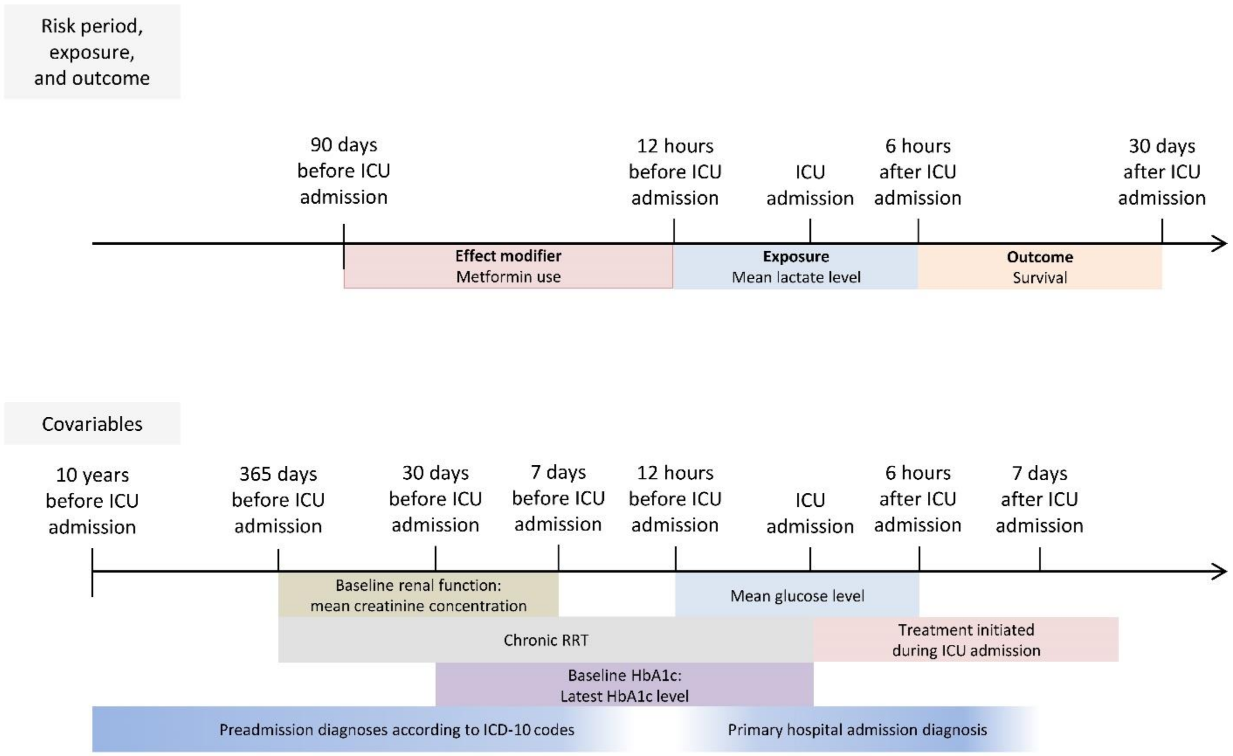


**Figure S1 – Schematic representation of the study design.**

**Additional file 1 -** Prognostic impact of elevated lactate levels on mortality in critically ill patients with and without preadmission metformin treatment: a Danish registry-based cohort study

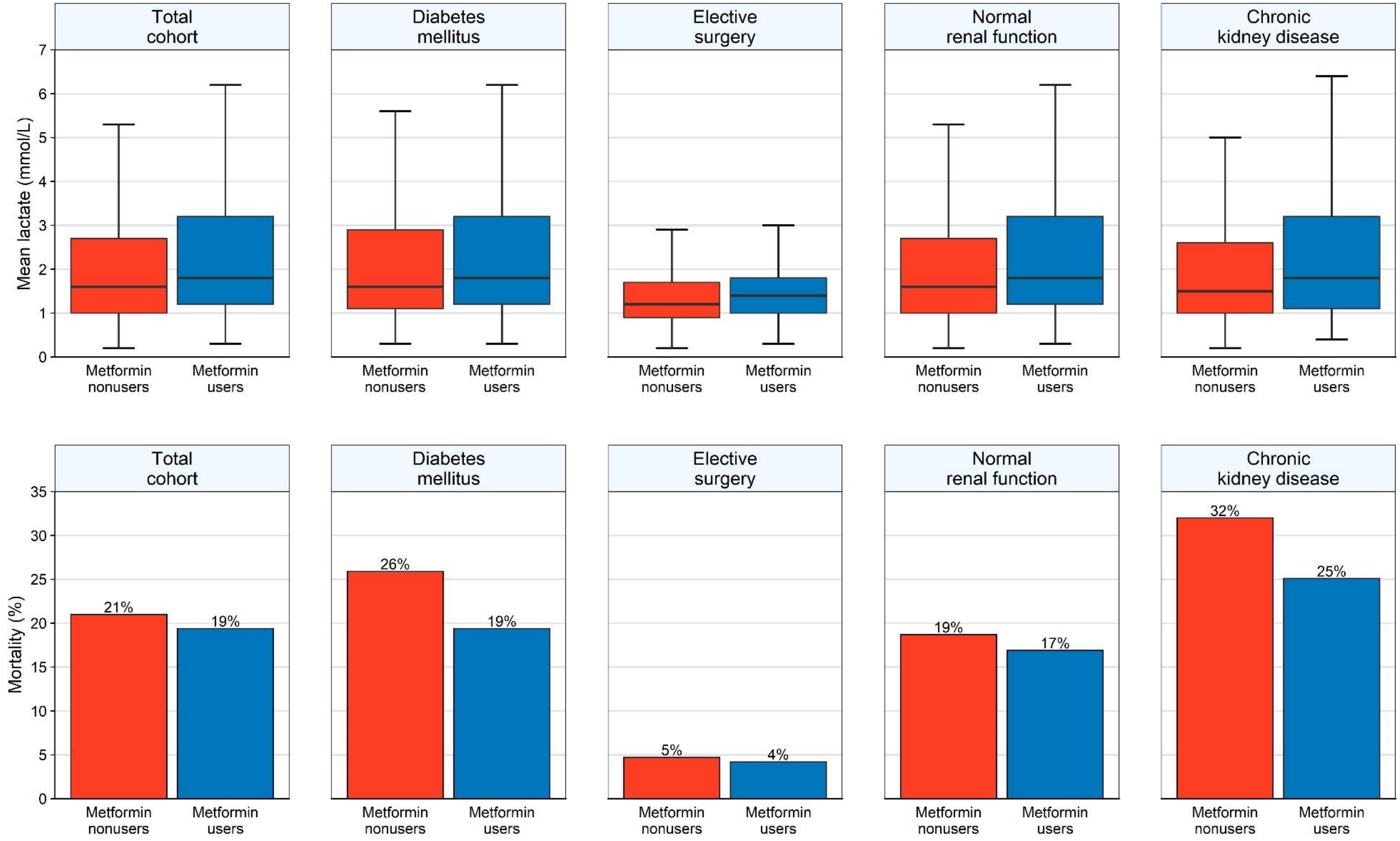


**Figure S2 – Tukey's boxplot of mean lactate levels and mortality rate for patients in the total cohort, among patients with diabetes mellitus, undergoing elective surgery, or with a normal renal function (eGFR ≥60 ml/min/1.72m$^2$) or chronic kidney disease (eGFR <60 ml/min/1.72m$^2$) one year before ICU admission.**

**Additional file 1 -** Prognostic impact of elevated lactate levels on mortality in critically ill patients with and without preadmission metformin treatment: a Danish registry-based cohort study

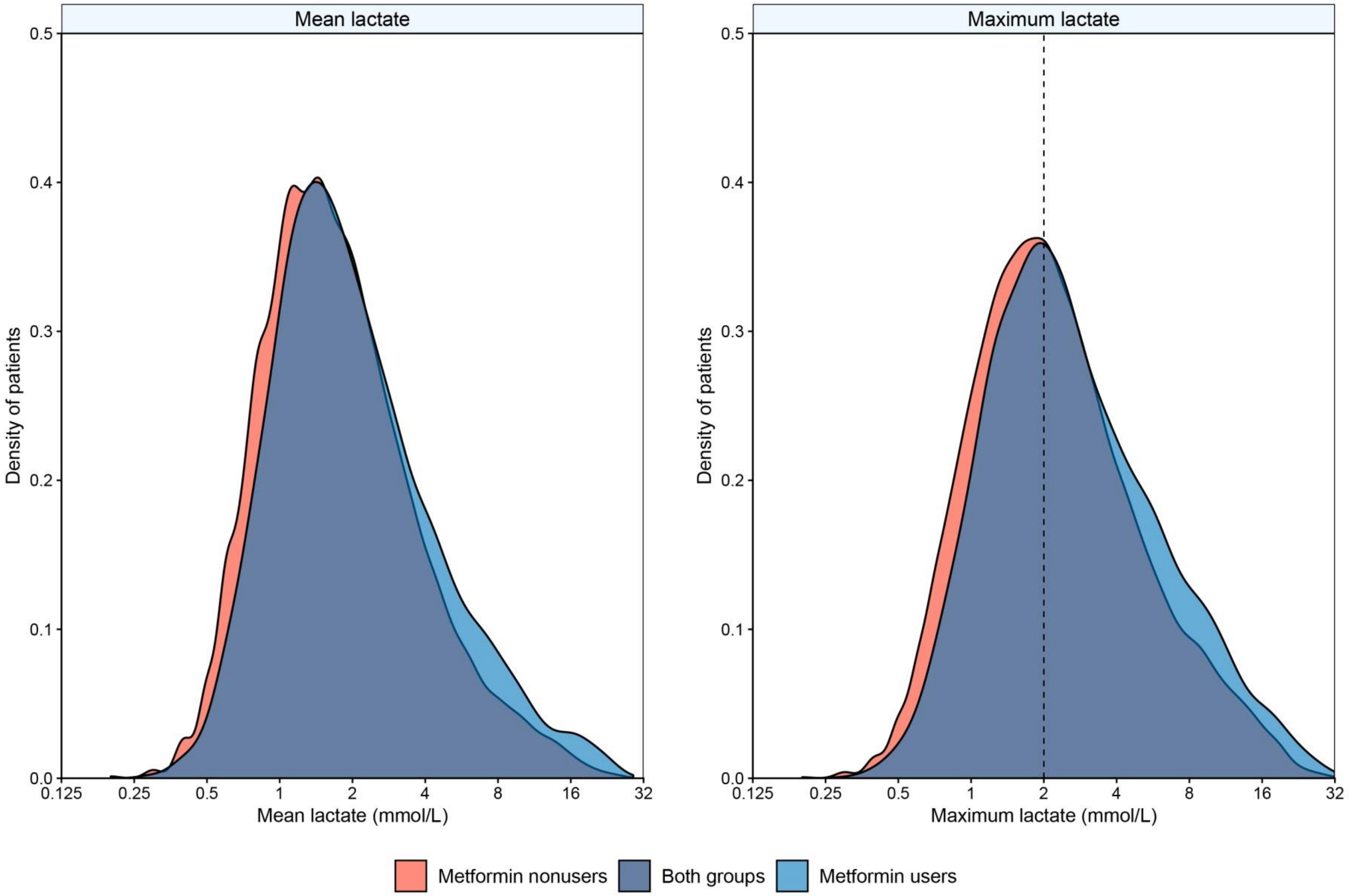


**Figure S3 – Distribution of metformin users and nonusers based on mean or maximum lactate level**
Both for mean lactate level and maximum lactate level around ICU admission, metformin users more often have higher lactate levels than nonusers, illustrated by the rightward shift of the curve. Therefore, more metformin users have a maximum lactate ≥2 mmol/L (dashed line), which is often used to identify severe critical illness, such as septic shock. The x-axis is logarithmically transformed because of the skewed data distribution.

**Additional file 1 -** Prognostic impact of elevated lactate levels on mortality in critically ill patients with and without preadmission metformin treatment: a Danish registry-based cohort study

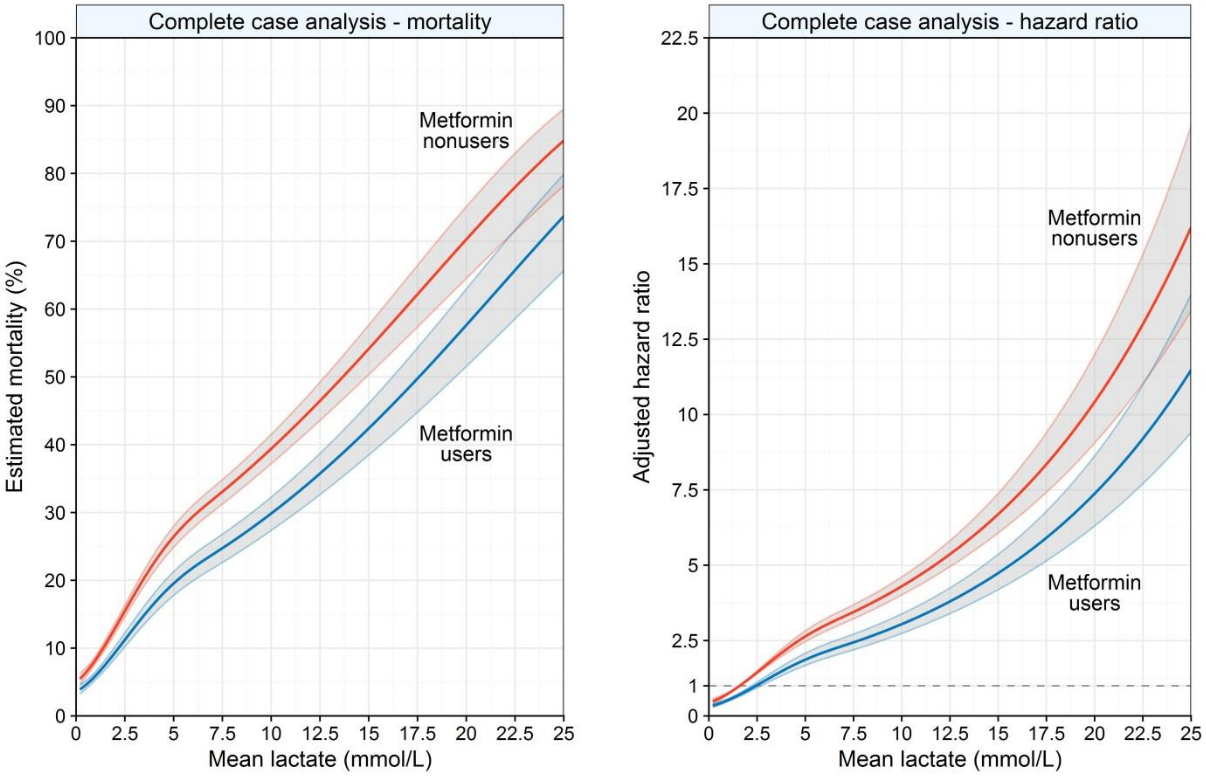


**Figure S4 - Association of lactate level with estimated 30-day mortality for metformin users and nonusers when restricted to complete cases**

Data were fit by a multivariable-adjusted Cox regression model based on restricted cubic splines constructed with four evenly spaced knots. A dataset restricted to complete cases was used for this analysis. The grey area represents the 95% confidence interval.

**Additional file 1 -** Prognostic impact of elevated lactate levels on mortality in critically ill patients with and without preadmission metformin treatment: a Danish registry-based cohort study

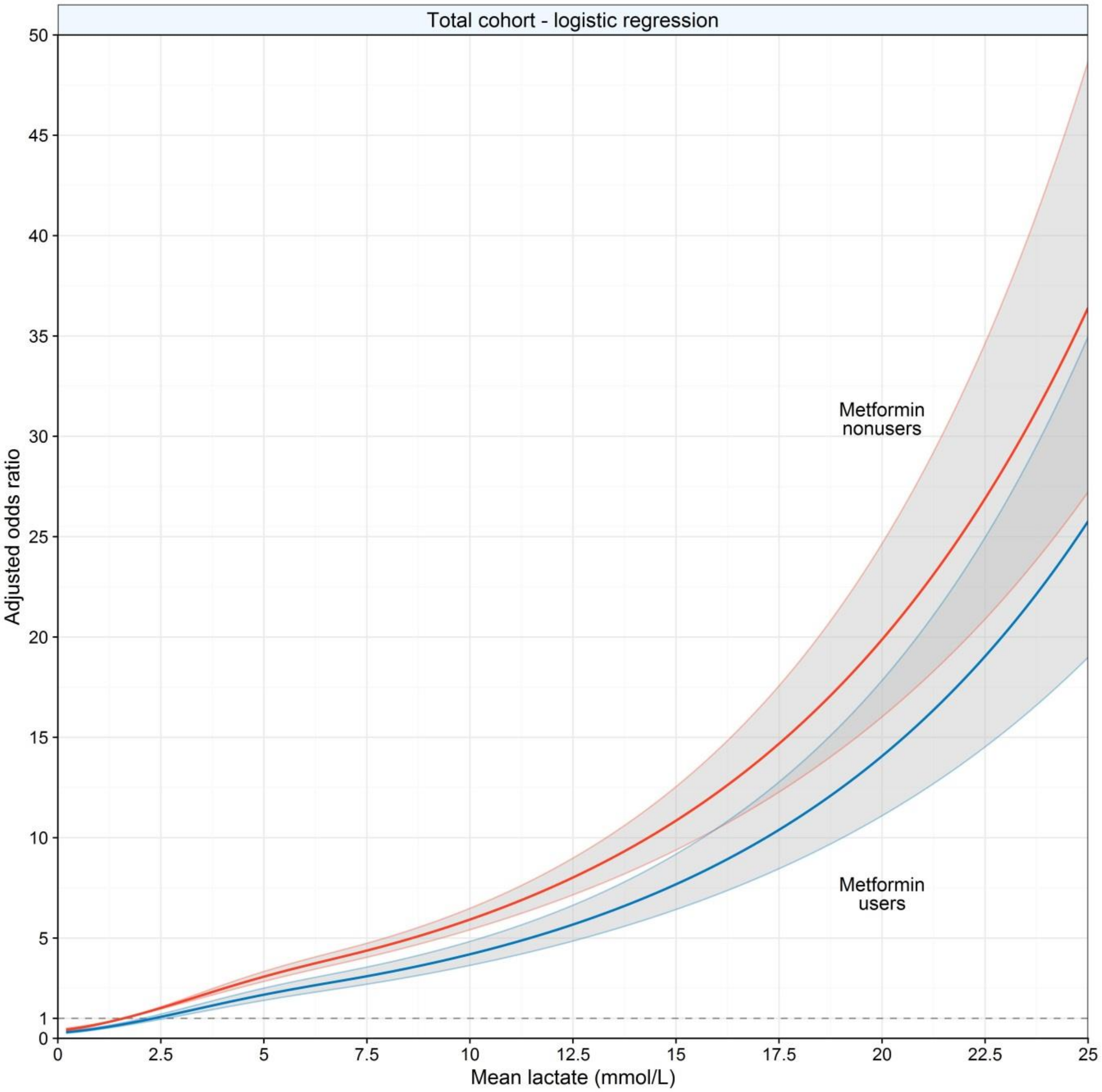


**Figure S5 – Association of mean lactate level with adjusted odds ratio for 30-day mortality for metformin users and nonusers in the total cohort.**
Data were fit by a multivariable-adjusted logistic regression model based on restricted cubic splines constructed with four evenly spaced knots. A multiple imputed dataset was used for this analysis. The grey area represents the 95% confidence interval.

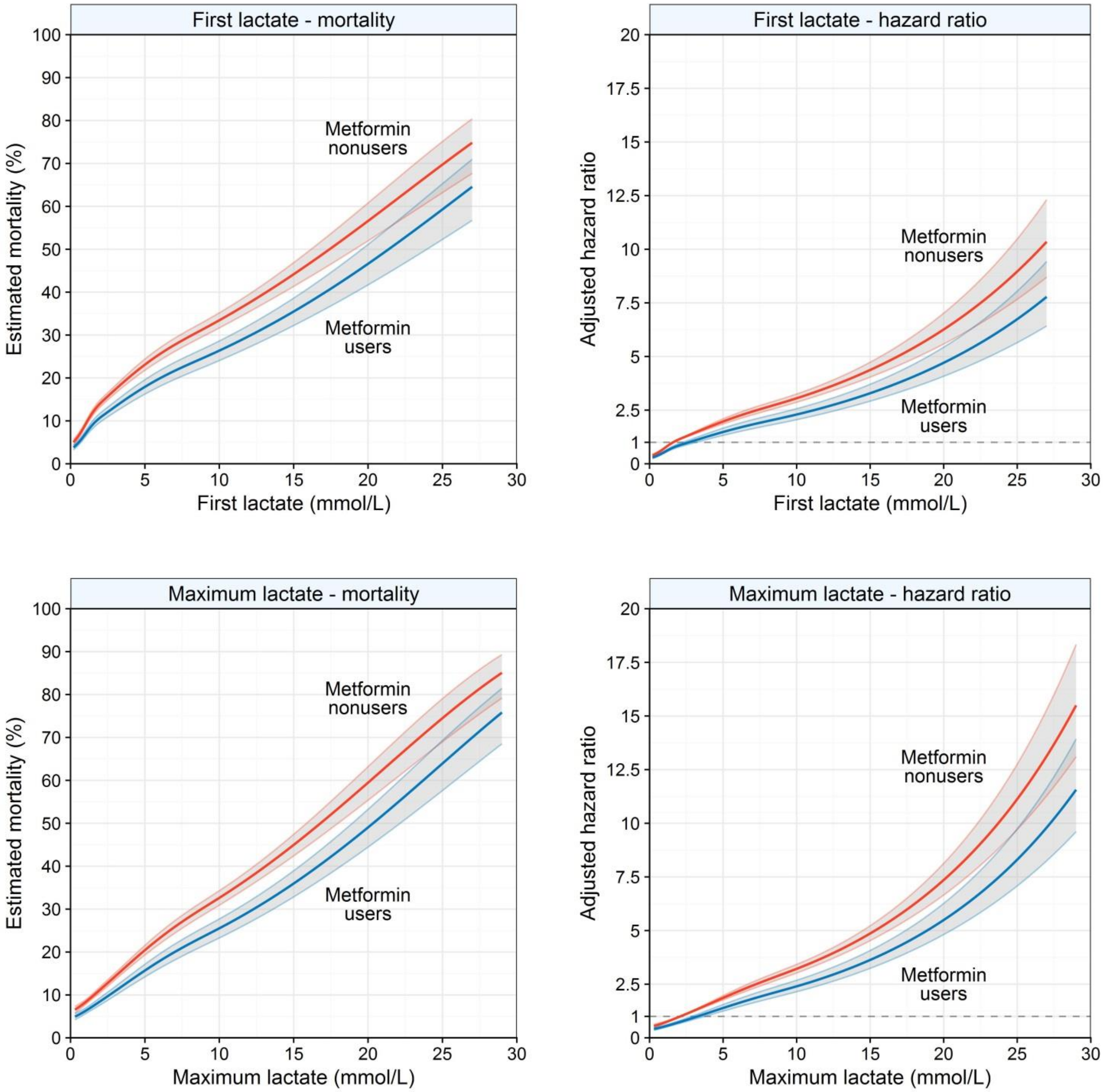


**Figure S6 – Association of first and maximum lactate level, respectively, measured within 12 hours before until 6 hours after ICU admission with estimated 30-day mortality and its corresponding adjusted hazard ratio for metformin users and nonusers in the total cohort.**

Data were fit by a multivariable-adjusted Cox regression model based on restricted cubic splines constructed with four evenly spaced knots. A multiple imputed dataset was used for this analysis. The grey area represents the 95% confidence interval.

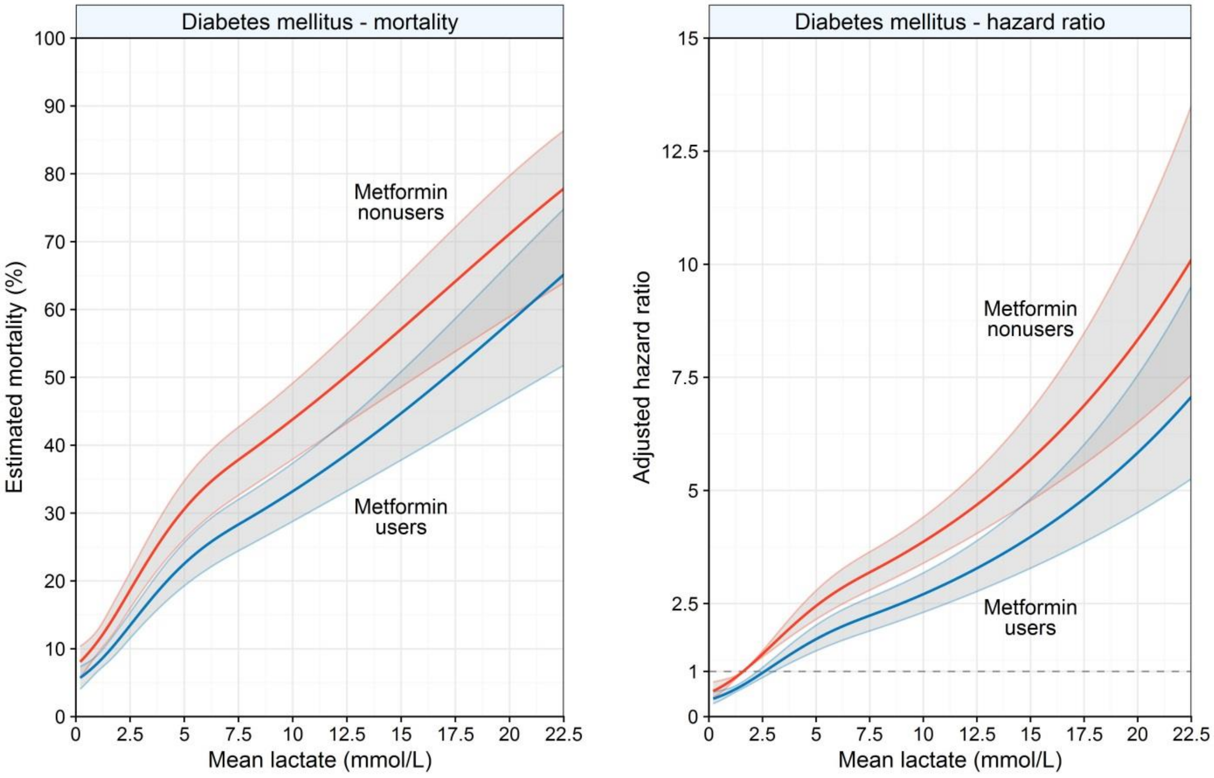


**Figure S7 – Association of lactate level with estimated 30-day mortality and its corresponding adjusted hazard ratio for metformin users and nonusers when including patients with diabetes mellitus.**
Data were fit by a multivariable-adjusted Cox regression model based on restricted cubic splines constructed with four evenly spaced knots. A multiple imputed dataset was used for this analysis. The grey area represents the 95% confidence interval.

**Additional file 1 -** Prognostic impact of elevated lactate levels on mortality in critically ill patients with and without preadmission metformin treatment: a Danish registry-based cohort study

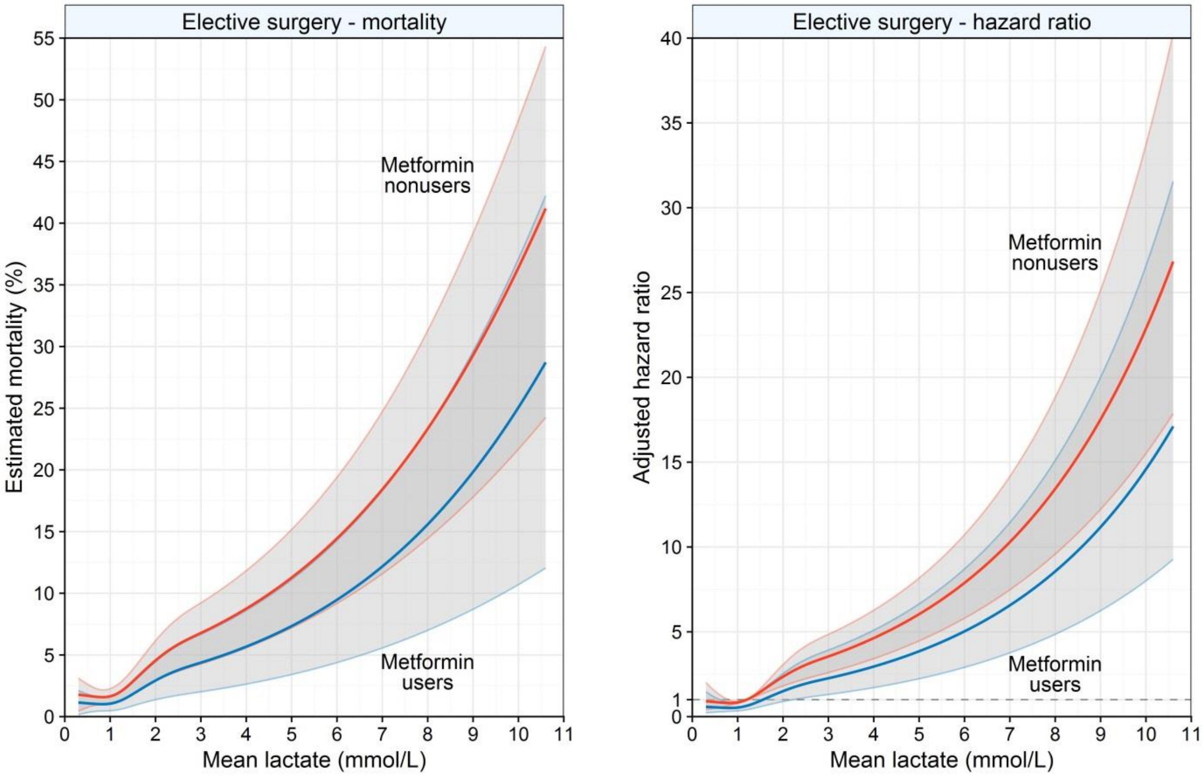


**Figure S8 – Association of lactate level with estimated 30-day mortality and its corresponding adjusted hazard ratio for metformin users and nonusers when only including patients undergoing elective surgery.**
Data were fit by a multivariable-adjusted Cox regression model based on restricted cubic splines constructed with four evenly spaced knots. A multiple imputed dataset was used for this analysis. The grey area represents the 95% confidence interval.

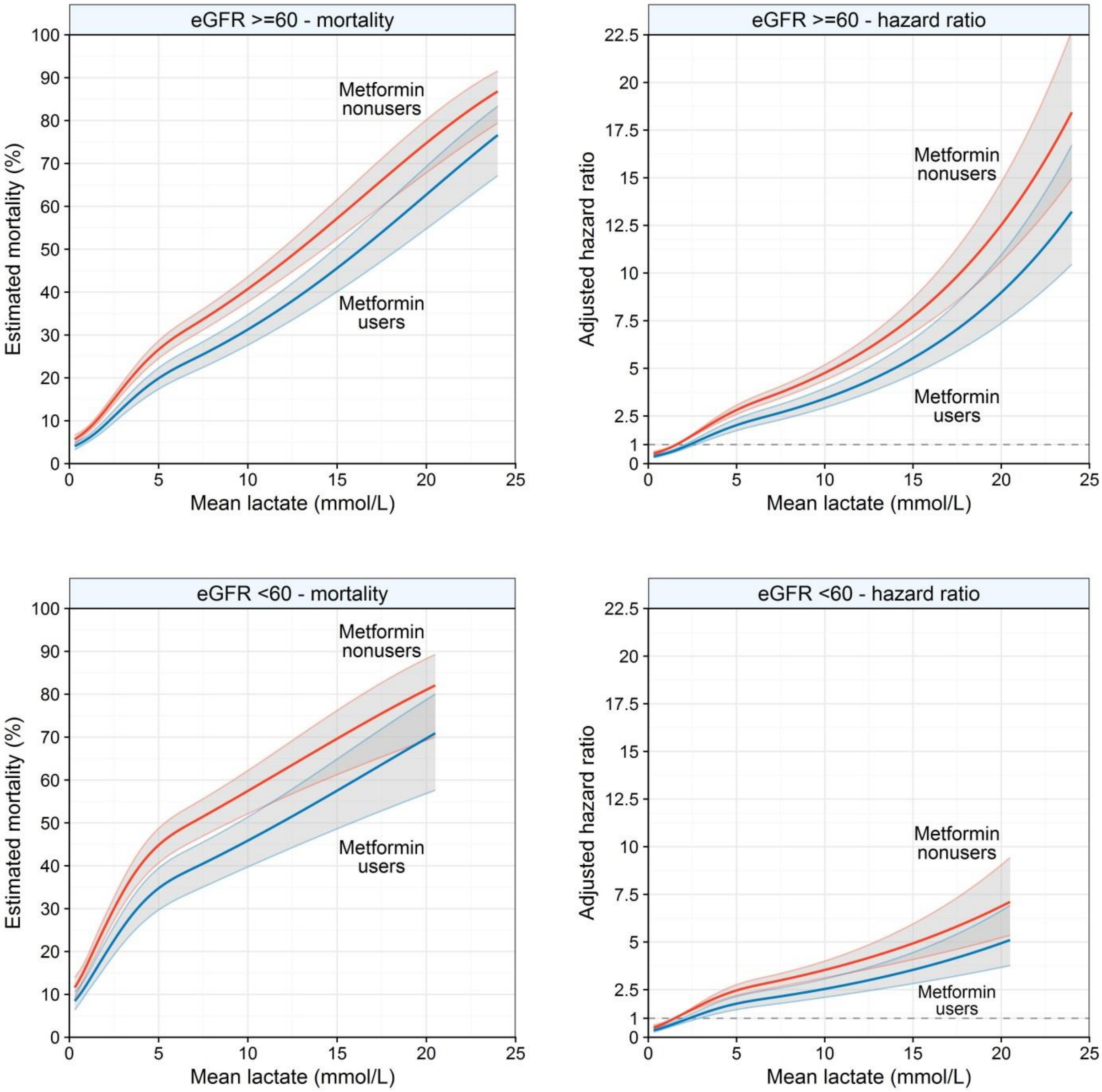


**Figure S9 – Association of lactate level with estimated 30-day mortality and its corresponding adjusted hazard ratio for metformin users and nonusers among patients with a normal renal function (eGFR ≥60 ml/min/1.72m$^2$) or chronic kidney disease (eGFR <60 ml/min/1.72m$^2$) one year before ICU admission.**

Data were fit by a multivariable-adjusted Cox regression model based on restricted cubic splines constructed with four evenly spaced knots. A multiple imputed dataset was used for this analysis. The grey area represents the 95% confidence interval.

**Additional file 1 -** Prognostic impact of elevated lactate levels on mortality in critically ill patients with and without preadmission metformin treatment: a Danish registry-based cohort study

**Table S1 – Characteristics of patients with and without available lactate measurements**

| **Characteristic** | **Total** | **Lactate available** | **Lactate not available** |
|---|---|---|---|
| No. of patients | 51,517 | 37,293 (72) | 14,224 (28) |
| Age, years | 66 [53-76] | 68 [56-77] | 63 [46-74] |
| Male | 28,866 | 21,244 (74) | 7622 (26) |
| **Year of admission** | | | |
| 2010 | 8043 | 4513 (56) | 3530 (44) |
| 2011 | 7313 | 4779 (65) | 2534 (35) |
| 2012 | 6900 | 4850 (70) | 2050 (30) |
| 2013 | 6516 | 4793 (74) | 1723 (26) |
| 2014 | 6484 | 5013 (77) | 1471 (23) |
| 2015 | 6438 | 5111 (79) | 1327 (21) |
| 2016 | 6244 | 5195 (83) | 1049 (17) |
| 2017 | 3579 | 3039 (85) | 540 (15) |
| **Charlson comorbidity index score** | | | |
| 0 | 20,562 | 13,769 (67) | 6793 (33) |
| 1-2 | 18,802 | 14,005 (74) | 4797 (26) |
| ≥3 | 12,153 | 9519 (78) | 2634 (22) |
| **Preadmission disease** | | | |
| Diabetes mellitus | 9623 | 7703 (80) | 1920 (20) |
| Myocardial infarction | 3493 | 863 (25) | 2630 (75) |
| Congestive heart failure | 4472 | 952 (21) | 3520 (79) |
| Peripheral vascular disease | 5242 | 1138 (22) | 4104 (78) |
| Cerebrovascular disease | 6834 | 1769 (26) | 5065 (74) |
| Dementia | 739 | 177 (24) | 562 (76) |
| Chronic pulmonary disease | 8324 | 1461 (18) | 6863 (72) |
| Connective tissue disease | 2301 | 609 (26) | 1692 (74) |
| Ulcer disease | 2781 | 616 (22) | 2165 (78) |
| Mild liver disease | 1685 | 309 (18) | 1376 (82) |
| Moderate to severe liver disease | 664 | 96 (14) | 568 (86) |
| Moderate to severe renal disease | 3157 | 2464 (78) | 693 (22) |
| Hemiplegia | 444 | 349 (79) | 95 (1) |
| Any tumor | 8219 | 5942 (72) | 2277 (28) |
| Metastatic solid tumor | 1952 | 1420 (73) | 532 (27) |
| Leukemia | 349 | 287 (82) | 62 (18) |
| Lymphoma | 801 | 645 (81) | 156 (19) |
| AIDS[a] | <70 | <50 (<75) | 19 (<25) |
| **Renal function during 1 year before ICU admission[b]** | | | |
| eGFR, ml/min/1.73m$^2$ | 81 [60-96] | 80 [59-95] | 84 [64-98] |
| ≥60 ml/min/1.73m$^2$ | 31,436 | 22,770 (72) | 8666 (28) |
| 45-60 ml/min/1.73m$^2$ | 4875 | 3773 (77) | 1102 (22) |

| | | | |
|---|---|---|---|
| 30-45 ml/min/1.73m$^2$ | 3041 | 2402 (79) | 639 (21) |
| <30 ml/min/1.73m$^2$ | 2155 | 1641 (76) | 514 (24) |
| Dialysis before ICU admission[a] | <500 | <350 (<70) | 144 (<30) |
| **Antihyperglycemic therapy** | | | |
| Metformin | 3961 | 3183 (80) | 778 (20) |
| Sulfonylureas | 1160 | 899 (76) | 261 (23) |
| Insulin | 2871 | 2343 (82) | 528 (18) |
| Other antihyperglycemic agents | 836 | 691 (83) | 145 (17) |
| HbA1c within 4 weeks before ICU admission, %[c] | 5.9 [5.5-6.7] | 5.9 [5.5-6.7] | 5.9 [5.4-6.6] |
| **ICU admission type[d]** | | | |
| Medical | 22,440 | 17,926 (80) | 4514 (20) |
| Emergency surgery | 15,287 | 10,683 (70) | 4604 (30) |
| Elective surgery | 8484 | 6193 (73) | 2291 (27) |
| SAPS-II score[e] | 39 [29-52] | 40 [30-52] | 37 [25-49] |
| **Primary diagnostic category** | | | |
| Cardiovascular | 11,903 | 8466 (71) | 3437 (29) |
| Respiratory, incl. pneumonia | 5918 | 5244 (89) | 674 (11) |
| Infection or sepsis, excl. pneumonia | 4331 | 3425 (79) | 906 (21) |
| Gastrointestinal and liver disease | 5498 | 4215 (77) | 1283 (23) |
| Neoplasms | 5879 | 3899 (66) | 1980 (34) |
| Trauma and poisoning | 7543 | 5014 (66) | 2529 (34) |
| Endocrinology | 1156 | 1013 (88) | 143 (12) |
| Other | 9289 | 6017 (65) | 3272 (13) |
| **Laboratory values 12h before until 6h after ICU admission** | | | |
| Mean glucose, mmol/L[f] | 7.9 [6.6-9.9] | 8.1 [6.7-10.1] | 7.0 [5.9-8.8] |
| **ICU treatment** | | | |
| Mechanical ventilation | 19,243 | 15,594 (81) | 3649 (19) |
| Inotropes or vasopressors | 15,760 | 13,081 (83) | 2679 (17) |
| Renal replacement therapy | 2369 | 1919 (81) | 450 (19) |
| ICU length of stay, days[h] | 1.0 [0.6-2.4] | 1.1 [0.7-2.8] | 0.8 [0.3-1.3] |
| Hospital length of stay, days | 10 [4-20] | 10 [5-20] | 8 [3-17] |
| **Outcome** | | | |
| 30-day mortality | 9455 | 7768 (82) | 1687 (18) |
| 90-day mortality | 11,767 | 9564 (81) | 2203 (19) |

Blood lactate level category based on quintiles and clinically relevant boundaries in mmol/L. Data are expressed as n (%) or median [IQR]. Percentage was calculated by row instead of by column.

[a] According to Danish regulation, frequencies smaller than five patients not being zero and the corresponding total number of patients are not reported. Because in other tables the total number of patients was not reported, we have omitted this number in the table as well.

[b] Data missing for 10,010 (19%) patients

[c] Data missing for 43,954 (85%) patients

[d] Data missing for 5306 (10%) patients

[e] Data missing for 38,453 (75%) patients

[f] Data missing for 10,052 (20%) patients

[h] Data missing for 5272 (10%) patients

**Additional file 1 -** Prognostic impact of elevated lactate levels on mortality in critically ill patients with and without preadmission metformin treatment: a Danish registry-based cohort study

**Table S2 – Individual components of the Charlson Comorbidity Index in the full cohort**

| **Characteristic** | **Total** | **<1.4** | **1.4 to 2.0** | **2.0 to 3.2** | **3.2 to 5.0** | **5.0 to 10.0** | **≥10** |
|---|---|---|---|---|---|---|---|
| No. of patients | 37,293 | 14,572 | 8705 | 6831 | 3694 | 2544 | 947 |
| Myocardial infarction | 2630 (7) | 1108 (8) | 621 (7) | 425 (6) | 251 (7) | 162 (6) | 63 (7) |
| Congestive heart failure | 3520 (9) | 1424 (10) | 837 (10) | 594 (9) | 341 (9) | 236 (9) | 88 (9) |
| Peripheral vascular disease | 4104 (11) | 1648 (11) | 998 (11) | 681 (10) | 400 (11) | 278 (11) | 99 (10) |
| Cerebrovascular disease | 5065 (14) | 2084 (14) | 1138 (13) | 876 (13) | 511 (14) | 333 (13) | 123 (13) |
| Dementia | 562 (2) | 204 (1) | 125 (1) | 107 (2) | 71 (2) | 37 (1) | 18 (2) |
| Chronic pulmonary disease | 6863 (18) | 3011 (21) | 1563 (18) | 1151 (17) | 642 (17) | 387 (15) | 109 (12) |
| Connective tissue disease | 1692 (5) | 638 (4) | 416 (5) | 306 (4) | 178 (5) | 112 (4) | 42 (4) |
| Ulcer disease | 2165 (6) | 883 (6) | 447 (5) | 390 (6) | 221 (6) | 158 (6) | 66 (7) |
| Mild liver disease | 1376 (4) | 347 (2) | 283 (3) | 264 (4) | 193 (5) | 193 (8) | 96 (10) |
| Moderate to severe liver disease | 568 (2) | 108 (1) | 119 (1) | 113 (2) | 92 (2) | 92 (4) | 44 (5) |
| Diabetes without end-organ failure | 4855 (13) | 1802 (12) | 1101 (13) | 877 (13) | 520 (14) | 407 (16) | 148 (16) |
| Diabetes with end-organ failure | 2698 (7) | 1025 (7) | 603 (7) | 472 (7) | 279 (8) | 235 (9) | 84 (9) |
| Moderate to severe renal disease | 2464 (7) | 1068 (7) | 522 (6) | 402 (6) | 259 (7) | 157 (6) | 56 (6) |
| Hemiplegia | 349 (1) | 161 (1) | 79 (1) | 56 (1) | 26 (1) | 18 (1) | 9 (1) |
| Any tumor | 5942 (16) | 2476 (17) | 1348 (15) | 1094 (16) | 597 (16) | 323 (13) | 104 (11) |
| Metastatic solid tumor | 1420 (4) | 492 (3) | 344 (4) | 307 (4) | 179 (5) | 70 (3) | 28 (3) |
| Leukemia | 287 (1) | 110 (1) | 65 (1) | 55 (1) | 29 (1) | 23 (1) | 5 (1) |
| Lymphoma | 645 (2) | 239 (2) | 140 (2) | 114 (2) | 71 (2) | 69 (3) | 12 (1) |
| AIDS[a] | <50 | 14 (0) | 11 (0) | 9 (0) | 5 (0) | <5 (0) | <5 (0) |

Blood lactate level category based on quintiles and clinically relevant boundaries in mmol/L. Data are expressed as n (%). Data are based on international classification of diseases codes only.

[a] According to Danish regulation, frequencies smaller than five patients not being zero and the corresponding total number of patients are not reported.

**Additional file 1 -** Prognostic impact of elevated lactate levels on mortality in critically ill patients with and without preadmission metformin treatment: a Danish registry-based cohort study

**Table S3 – Characteristics of metformin nonusers and metformin users**

| **Characteristic** | **Total** | **Metformin nonusers** | **Metformin users** |
|---|---|---|---|
| No. of patients | 37,293 | 34,110 | 3183 |
| Age, years | 68 [56-77] | 67 [55-77] | 70 [63-76] |
| Male | 21,244 (57) | 19,287 (57) | 1957 (61) |
| **Charlson comorbidity index score** | | | |
| 0 | 13,769 (37) | 13,241 (39) | 528 (17) |
| 1-2 | 14,005 (38) | 12,717 (37) | 1288 (40) |
| ≥3 | 9519 (26) | 8152 (24) | 1367 (43) |
| **Preadmission disease**[a] | | | |
| Myocardial infarction | 2630 (7) | 2334 (7) | 296 (9) |
| Congestive heart failure | 3520 (9) | 3050 (9) | 470 (15) |
| Peripheral vascular disease | 4104 (11) | 3638 (11) | 466 (15) |
| Cerebrovascular disease | 5065 (14) | 4541 (13) | 524 (16) |
| Dementia | 562 (2) | 502 (1) | 60 (2) |
| Chronic pulmonary disease | 6863 (18) | 6215 (18) | 648 (20) |
| Connective tissue disease | 1692 (5) | 1555 (5) | 137 (4) |
| Ulcer disease | 2165 (6) | 1981 (6) | 184 (6) |
| Mild liver disease | 1376 (4) | 1289 (4) | 87 (3) |
| Moderate to severe liver disease | 568 (2) | 529 (2) | 39 (1) |
| Diabetes without end-organ failure | 4855 (13) | 2993 (9) | 1862 (58) |
| Diabetes with end-organ failure | 2698 (7) | 1804 (5) | 894 (28) |
| Moderate to severe renal disease | 2464 (7) | 2298 (7) | 166 (5) |
| Hemiplegia | 349 (1) | 328 (1) | 21 (1) |
| Any tumor | 5942 (16) | 5425 (16) | 517 (16) |
| Metastatic solid tumor | 1420 (4) | 1336 (4) | 84 (3) |
| Leukemia | 287 (1) | 271 (1) | 16 (1) |
| Lymphoma | 645 (2) | 606 (2) | 39 (1) |
| AIDS[b] | <45 | 41 (0) | <5 |
| **Renal function during 1 year before ICU admission**[c] | | | |
| eGFR, ml/min/1.73m$^2$ | 80 [59-95] | 81 [59-96] | 77 [58-92] |
| ≥60 ml/min/1.73m$^2$ | 22,770 (74) | 20,539 (75) | 2231 (73) |
| 45-60 ml/min/1.73m$^2$ | 3773 (12) | 3289 (12) | 484 (16) |
| 30-45 ml/min/1.73m$^2$ | 2402 (8) | 2142 (8) | 260 (9) |

| | | | |
|---|---|---|---|
| <30 ml/min/1.73m$^2$ | 1641 (5) | 1576 (6) | 65 (2) |
| Dialysis before ICU admission[b] | <350 | 343 (1) | <5 |
| **Antihyperglycemic therapy** | | | |
| Sulfonylureas | 899 (2) | 410 (1) | 489 (15) |
| Insulin | 2343 (6) | 1652 (5) | 691 (22) |
| Other antihyperglycemic agents | 691 (2) | 281 (1) | 410 (13) |
| HbA1c within four weeks before ICU admission, %[d] | 5.9 [5.5-6.7] | 5.8 [5.4-6.4] | 6.9 [6.3-7.8] |
| **ICU admission type[e]** | | | |
| Medical | 17,926 (52) | 16,287 (51) | 1639 (54) |
| Emergency surgery | 10,683 (31) | 9974 (31) | 709 (23) |
| Elective surgery | 6193 (18) | 5521 (17) | 672 (22) |
| SAPS-II score[f] | 40 [30-52] | 40 [30-52] | 42 [31-54] |
| **Primary diagnostic category** | | | |
| Cardiovascular | 8466 (23) | 7630 (22) | 836 (26) |
| Respiratory, incl. pneumonia | 5244 (14) | 4721 (14) | 523 (16) |
| Infection or sepsis, excl. pneumonia | 3425 (9) | 3123 (9) | 302 (9) |
| Gastrointestinal and liver disease | 4215 (11) | 3905 (11) | 310 (10) |
| Neoplasms | 3899 (10) | 3591 (11) | 308 (10) |
| Trauma and poisoning | 5014 (13) | 4748 (14) | 266 (8) |
| Endocrinology | 1013 (3) | 872 (3) | 141 (4) |
| Other | 6017 (16) | 5520 (16) | 497 (16) |
| **Laboratory values 12h before until 6h after ICU admission** | | | |
| First lactate, mmol/L | 1.6 [1.0-3.0] | 1.6 [1.0-2.9] | 1.8 [1.1-3.7] |
| Mean lactate, mmol/L | 1.6 [1.1-2.8] | 1.6 [1.0-2.7] | 1.8 [1.2-3.2] |
| Maximum lactate, mmol/L | 2.1 [1.3-3.7] | 2.1 [1.3-3.7] | 2.4 [1.5-4.5] |
| No. of lactate measurements | 4 [2-6] | 4 [2-6] | 4 [2-6] |
| Mean glucose, mmol/L[g] | 8.1 [6.7-10.1] | 7.9 [6.6-9.8] | 10.2 [8.2-13.1] |
| **ICU treatment** | | | |
| Mechanical ventilation | 15,594 (42) | 14,205 (42) | 1389 (44) |
| Inotropes or vasopressors | 13,081 (35) | 11,824 (35) | 1257 (39) |
| Renal replacement therapy | 1919 (5) | 1697 (5) | 222 (7) |
| ICU length of stay, days[h] | 1.1 [0.7-2.8] | 1.0 [0.7-2.8] | 1.3 [0.8-3.1] |
| Hospital length of stay, days | 10 [5-20] | 10 [5-21] | 11 [6-20] |

**Additional file 1 -** Prognostic impact of elevated lactate levels on mortality in critically ill patients with and without preadmission metformin treatment: a Danish registry-based cohort study

| **Outcome** | | | |
|---|---|---|---|
| 30-day mortality | 7768 (21) | 7150 (21) | 618 (19) |
| 90-day mortality | 9564 (26) | 8786 (26) | 778 (24) |

Data are expressed as n (%) or median [IQR].

[a] Based on international classification of diseases codes only
[b] According to Danish regulation, frequencies smaller than five patients not being zero and the corresponding total number of patients are not reported.

[c] Data missing for 6707 (18%) patients

[d] Data missing for 31,373 (84%) patients

[e] Data missing for 2491 (7%) patients

[f] Data missing for 25,932 (70%) patients

[g] Data missing for 807 (2%) patients

[h] Data missing for 2885 (8%) patients

**Additional file 1 -** Prognostic impact of elevated lactate levels on mortality in critically ill patients with and without preadmission metformin treatment: a Danish registry-based cohort study

**Table S4 – Thirty-day mortality, hazard ratios, and the relative excess risk due to interaction by lactate category for metformin nonusers and users when restricting to complete cases**

| | **Events / no. at risk** | | **Crude HR (95% CI)** | | **Adjusted HR (95% CI)[a]** | | **RERI (95% CI)[b]** |
|---|---|---|---|---|---|---|---|
| Lactate (mmol/L) | Metformin nonusers | Metformin users | Metformin nonusers | Metformin users | Metformin nonusers | Metformin users | |
| <1.4 | 1784 / 13,525 (13%) | 136 / 1047 (13%) | Reference | 0.98 (0.82 to 1.16) | Reference | 0.88 (0.74 to 1.06) | |
| 1.4 to 2.0 | 1400 / 7947 (18%) | 114 / 758 (15%) | 1.37 (1.28 to 1.47) | 1.15 (0.95 to 1.39) | 1.44 (1.34 to 1.56) | 1.10 (0.90 to 1.33) | -0.23 (-0.51 to 0.05) |
| 2.0 to 3.2 | 1422 / 6235 (23%) | 116 / 596 (19%) | 1.84 (1.72 to 1.97) | 1.53 (1.27 to 1.85) | 2.03 (1.88 to 2.19) | 1.46 (1.20 to 1.77) | -0.46 (-0.80 to -0.11) |
| 3.2 to 5.0 | 1082 / 3341 (32%) | 84 / 353 (24%) | 2.80 (2.60 to 3.02) | 1.90 (1.53 to 2.36) | 2.98 (2.74 to 3.23) | 1.84 (1.47 to 2.30) | -1.02 (-1.50 to -0.54) |
| 5.0 to 10.0 | 964 / 2244 (43%) | 105 / 300 (35%) | 4.17 (3.86 to 4.51) | 3.16 (2.59 to 3.84) | 4.63 (4.25 to 5.05) | 2.90 (2.36 to 3.56) | -1.61 (-2.31 to -0.92) |
| ≥10.0 | 498 / 818 (61%) | 63 / 129 (49%) | 7.60 (6.88 to 8.39) | 5.20 (4.04 to 6.69) | 8.93 (7.97 to 10.00) | 5.06 (3.89 to 6.58) | -3.75 (-5.36 to -2.14) |

Hazard ratio (HR) with 95% confidence interval (95% CI) was computed using Cox proportional regression analysis based on a dataset restricted to complete cases.

[a] Adjusted for age, gender, mean of all creatinine measurements 1 year before ICU admission, and previous diagnosis of myocardial infarction, congestive heart failure, peripheral artery disease, cerebrovascular disease, dementia, connective tissue disease, peptic ulcer disease, chronic pulmonary disease, mild to severe liver disease, any tumor, metastatic solid tumor, leukemia, and lymphoma, respectively, 10 years before ICU admission. Codes used for each variable are stated in Appendix 1.

[b] Relative excess risk due to interaction (RERI) quantifies interaction on an additive scale, which approaches zero in the absence of interaction.

**Additional file 1 -** Prognostic impact of elevated lactate levels on mortality in critically ill patients with and without preadmission metformin treatment: a Danish registry-based cohort study

**Table S5 – Thirty-day mortality, hazard ratios, and the relative excess risk due to interaction by lactate category for metformin nonusers and users when correcting for number of lactate measurements**

| | **Events / no. at risk** | | **Crude HR (95% CI)** | | **Adjusted HR (95% CI)[a]** | | **RERI (95% CI)[b]** |
|---|---|---|---|---|---|---|---|
| Lactate (mmol/L) | Metformin nonusers | Metformin users | Metformin nonusers | Metformin users | Metformin nonusers | Metformin users | |
| <1.4 | 1784 / 13,525 (13%) | 136 / 1047 (13%) | Reference | 0.98 (0.82 to 1.16) | Reference | 0.95 (0.79 to 1.13) | |
| 1.4 to 2.0 | 1400 / 7947 (18%) | 114 / 758 (15%) | 1.37 (1.28 to 1.47) | 1.15 (0.95 to 1.39) | 1.44 (1.34 to 1.55) | 1.13 (0.93 to 1.37) | -0.26 (-0.53 to 0.02) |
| 2.0 to 3.2 | 1422 / 6235 (23%) | 116 / 596 (19%) | 1.84 (1.72 to 1.97) | 1.53 (1.27 to 1.85) | 1.98 (1.85 to 2.13) | 1.51 (1.25 to 1.83) | -0.42 (-0.76 to -0.08) |
| 3.2 to 5.0 | 1082 / 3341 (32%) | 84 / 353 (24%) | 2.80 (2.60 to 3.02) | 1.90 (1.53 to 2.36) | 2.95 (2.74 to 3.19) | 1.85 (1.47 to 2.31) | -1.05 (-1.53 to -0.58) |
| 5.0 to 10.0 | 964 / 2244 (43%) | 105 / 300 (35%) | 4.17 (3.86 to 4.51) | 3.16 (2.59 to 3.84) | 4.52 (4.18 to 4.90) | 3.06 (2.50 to 3.74) | -1.41 (-2.10 to -0.73) |
| ≥10.0 | 498 / 818 (61%) | 63 / 129 (49%) | 7.60 (6.88 to 8.39) | 5.20 (4.04 to 6.69) | 9.20 (8.32 to 10.18) | 5.45 (4.22 to 7.04) | -3.69 (-5.31 to -2.08) |

Hazard ratio (HR) with 95% confidence interval (95% CI) was computed using Cox proportional regression analysis based on a dataset restricted to complete cases.

[a] Adjusted for age, gender, last HbA1c measurement within 4 weeks before ICU admission, mean of all creatinine measurements 1 year before ICU admission, number of lactate measurements, and previous diagnosis of myocardial infarction, congestive heart failure, peripheral artery disease, cerebrovascular disease, dementia, connective tissue disease, peptic ulcer disease, chronic pulmonary disease, mild to severe liver disease, any tumor, metastatic solid tumor, leukemia, and lymphoma, respectively, 10 years before ICU admission. Codes used for each variable are stated in Appendix 1.

[b] Relative excess risk due to interaction (RERI) quantifies interaction on an additive scale, which approaches zero in the absence of interaction.

**Additional file 1 -** Prognostic impact of elevated lactate levels on mortality in critically ill patients with and without preadmission metformin treatment: a Danish registry-based cohort study

**Table S6 – Thirty-day mortality, odds ratios, and the relative excess risk due to interaction by lactate category for metformin nonusers and users**

| | **Events / no. at risk** | | **Crude OR (95% CI)** | | **Adjusted OR (95% CI)[a]** | | **RERI (95% CI)[b]** |
|---|---|---|---|---|---|---|---|
| Lactate (mmol/L) | Metformin nonusers | Metformin users | Metformin nonusers | Metformin users | Metformin nonusers | Metformin users | |
| <1.4 | 1784 / 13,525 (13%) | 136 / 1047 (13%) | Reference | 0.98 (0.81 to 1.18) | Reference | 0.94 (0.79 to 1.16) | |
| 1.4 to 2.0 | 1400 / 7947 (18%) | 114 / 758 (15%) | 1.41 (1.30 to 1.52) | 1.17 (0.94 to 1.42) | 1.53 (1.41 to 1.66) | 1.17 (0.95 to 1.46) | -0.30 ( -0.62 to 0.01) |
| 2.0 to 3.2 | 1422 / 6235 (23%) | 116 / 596 (19%) | 1.94 (1.80 to 2.10) | 1.59 (1.29 to 1.95) | 2.22 (2.05 to 2.42) | 1.60 (1.31 to 2.03) | -0.56 ( -0.97 to -0.15) |
| 3.2 to 5.0 | 1082 / 3341 (32%) | 84 / 353 (24%) | 3.15 (2.89 to 3.44) | 2.06 (1.59 to 2.63) | 3.62 (3.30 to 3.98) | 2.05 (1.61 to 2.71) | -1.51 ( -2.14 to -0.87) |
| 5.0 to 10.0 | 964 / 2244 (43%) | 105 / 300 (35%) | 4.96 (4.50 to 5.46) | 3.54 (2.77 to 4.50) | 6.04 (5.45 to 6.72) | 3.69 (2.90 to 4.82) | -2.29 ( -3.38 to -1.19) |
| ≥10.0 | 498 / 818 (61%) | 63 / 129 (49%) | 10.24 (8.83 to 11.90) | 6.28 (4.43 to 8.91) | 15.80 (13.41 to 18.56) | 7.37 (5.19 to 10.73) | -8.37 (-12.00 to -4.73) |

Odds ratio (OR) with 95% confidence interval (95% CI) was computed using logistic regression analysis based on a multiple imputed dataset.

[a] Adjusted for age, gender, last HbA1c measurement within 4 weeks before ICU admission, mean of all creatinine measurements 1 year before ICU admission, and previous diagnosis of myocardial infarction, congestive heart failure, peripheral artery disease, cerebrovascular disease, dementia, connective tissue disease, peptic ulcer disease, chronic pulmonary disease, mild to severe liver disease, any tumor, metastatic solid tumor, leukemia, and lymphoma, respectively, 10 years before ICU admission. Codes used for each variable are stated in Appendix 1.

**[b]** Relative excess risk due to interaction (RERI) quantifies interaction on an additive scale, which approaches zero in the absence of interaction.

**Additional file 1 -** Prognostic impact of elevated lactate levels on mortality in critically ill patients with and without preadmission metformin treatment: a Danish registry-based cohort study

**Table S7 – Characteristics of patients with diabetes mellitus stratified by lactate level category**

| **Characteristic** | **Total** | **<1.4** | **1.4 to 2.0** | **2.0 to 3.2** | **3.2 to 5.0** | **5.0 to 10.0** | **≥10** |
|---|---|---|---|---|---|---|---|
| No. of patients | 7703 | 2790 | 1787 | 1404 | 812 | 673 | 237 |
| Age, years | 70 [62-78] | 70 [62-77] | 70 [61-78] | 70 [60-78] | 70 [63-78] | 70 [61-77] | 68 [62-76] |
| Male | 4637 (60) | 1677 (60) | 1080 (60) | 847 (60) | 478 (59) | 416 (62) | 139 (59) |
| **Charlson comorbidity index score** | | | | | | | |
| 0 | 915 (12) | 297 (11) | 223 (12) | 177 (13) | 90 (11) | 92 (14) | 36 (15) |
| 1-2 | 2904 (38) | 1015 (36) | 710 (40) | 535 (38) | 304 (37) | 264 (39) | 76 (32) |
| ≥3 | 3884 (50) | 1478 (53) | 854 (48) | 692 (49) | 418 (51) | 317 (47) | 125 (53) |
| **Preadmission disease[a]** | | | | | | | |
| Myocardial infarction | 875 (11) | 338 (12) | 215 (12) | 152 (11) | 71 (9) | 74 (11) | 25 (11) |
| Congestive heart failure | 1364 (18) | 522 (19) | 308 (17) | 251 (18) | 130 (16) | 108 (16) | 45 (19) |
| Peripheral vascular disease | 1309 (17) | 490 (18) | 300 (17) | 241 (17) | 136 (17) | 100 (15) | 42 (18) |
| Cerebrovascular disease | 1360 (18) | 510 (18) | 304 (17) | 224 (16) | 154 (19) | 132 (20) | 36 (15) |
| Dementia[b] | <145 | 49 (2) | 34 (2) | 22 (2) | 20 (2) | 12 (2) | <5 |
| Chronic pulmonary disease | 1728 (22) | 706 (25) | 388 (22) | 299 (21) | 180 (22) | 121 (18) | 34 (14) |
| Connective tissue disease | 400 (5) | 131 (5) | 87 (5) | 75 (5) | 53 (7) | 39 (6) | 15 (6) |
| Ulcer disease | 553 (7) | 197 (7) | 123 (7) | 111 (8) | 61 (8) | 44 (7) | 17 (7) |
| Mild liver disease | 314 (4) | 75 (3) | 50 (3) | 64 (5) | 45 (6) | 52 (8) | 28 (12) |
| Moderate to severe liver disease | 147 (2) | 28 (1) | 22 (1) | 29 (2) | 27 (3) | 32 (5) | 9 (4) |
| Diabetes without end-organ failure | 4855 (63) | 1802 (65) | 1101 (62) | 877 (62) | 520 (64) | 407 (60) | 148 (62) |
| Diabetes with end-organ failure | 2698 (35) | 1025 (37) | 603 (34) | 472 (34) | 279 (34) | 235 (35) | 84 (35) |
| Moderate to severe renal disease | 967 (13) | 421 (15) | 196 (11) | 154 (11) | 98 (12) | 67 (10) | 31 (13) |
| Hemiplegia[b] | <50 (1) | 14 (1) | 15 (1) | 11 (1) | <5 | <5 | <5 |
| Any tumor | 1244 (16) | 495 (18) | 276 (15) | 217 (15) | 140 (17) | 84 (12) | 32 (14) |
| Metastatic solid tumor | 250 (3) | 87 (3) | 54 (3) | 43 (3) | 34 (4) | 21 (3) | 11 (5) |
| Leukemia[b] | <55 (1) | 15 (1) | 15 (1) | 9 (1) | <5 | 7 (1) | <5 |
| Lymphoma | 127 (2) | 36 (1) | 31 (2) | 26 (2) | 14 (2) | 16 (2) | 4 (2) |
| AIDS[b] | | | | | | | |
| **Renal function during 1 year before ICU admission[c]** | | | | | | | |
| eGFR, ml/min/1.73m$^2$ | 71 [48-91] | 69 [45-90] | 74 [50-92] | 73 [51-92] | 71 [50-91] | 72 [48-91] | 72 [50-93] |
| ≥60 ml/min/1.73m$^2$ | 4568 (63) | 1579 (60) | 1117 (66) | 834 (64) | 490 (65) | 406 (64) | 142 (65) |

| | | | | | | | |
|---|---|---|---|---|---|---|---|
| 45-60 ml/min/1.73m$^2$ | 1138 (16) | 425 (16) | 234 (14) | 230 (18) | 116 (15) | 98 (15) | 35 (16) |
| 30-45 ml/min/1.73m$^2$ | 930 (13) | 347 (13) | 214 (13) | 155 (12) | 96 (13) | 90 (14) | 28 (13) |
| <30 ml/min/1.73m$^2$ | 627 (9) | 301 (11) | 125 (7) | 89 (7) | 55 (7) | 42 (7) | 15 (7) |
| Dialysis before ICU admission[b] | <135 (2) | 57 (2) | 28 (2) | 22 (2) | 17 (2) | 7 (1) | <5 |
| **Antihyperglycemic therapy** | | | | | | | |
| Metformin | 3179 (41) | 1047 (38) | 756 (42) | 595 (42) | 352 (43) | 300 (45) | 129 (54) |
| Sulfonylureas | 899 (12) | 309 (11) | 233 (13) | 150 (11) | 104 (13) | 72 (11) | 31 (13) |
| Insulin | 2343 (30) | 853 (31) | 551 (31) | 428 (30) | 236 (29) | 220 (33) | 55 (23) |
| Other antihyperglycemic agents | 691 (9) | 206 (7) | 158 (9) | 131 (9) | 88 (11) | 72 (11) | 36 (15) |
| HbA1c within 4 weeks before ICU admission, %[d] | 6.9 [6.4-8.0] | 6.8 [6.3-7.8] | 6.9 [6.4-8.1] | 7.1 [6.4-8.3] | 6.9 [6.3-8.2] | 7.0 [6.4-8.2] | 7.0 [6.4-7.7] |
| **ICU admission type[e]** | | | | | | | |
| Medical | 4186 (57) | 1316 (50) | 876 (52) | 844 (63) | 528 (69) | 454 (71) | 168 (74) |
| Emergency surgery | 1822 (25) | 638 (24) | 412 (24) | 342 (25) | 208 (27) | 167 (26) | 55 (24) |
| Elective surgery[b] | <1305 (18) | 690 (26) | 400 (24) | 156 (12) | 33 (4) | 19 (3) | <5 |
| SAPS-II score[f] | 42 [31-54] | 37 [27-47] | 38 [29-49] | 42 [33-54] | 47 [37-59] | 56 [44-69] | 64 [52-79] |
| **Primary diagnostic category** | | | | | | | |
| Cardiovascular | 1829 (24) | 698 (25) | 440 (25) | 272 (19) | 164 (20) | 185 (27) | 70 (30) |
| Respiratory, incl. pneumonia | 1222 (16) | 520 (19) | 283 (16) | 222 (16) | 117 (14) | 66 (10) | 14 (6) |
| Infection or sepsis, excl. pneumonia | 836 (11) | 221 (8) | 193 (11) | 187 (13) | 128 (16) | 89 (13) | 18 (8) |
| Gastrointestinal and liver disease | 808 (10) | 277 (10) | 174 (10) | 157 (11) | 101 (12) | 64 (10) | 35 (15) |
| Neoplasms[b] | <670 (9) | 305 (11) | 175 (10) | 104 (7) | 44 (5) | 35 (5) | <5 |
| Trauma and poisoning | 622 (8) | 229 (8) | 156 (9) | 121 (9) | 62 (8) | 40 (6) | 14 (6) |
| Endocrinology | 583 (8) | 175 (6) | 124 (7) | 148 (11) | 56 (7) | 61 (9) | 19 (8) |
| Other | 1136 (15) | 365 (13) | 242 (14) | 193 (14) | 140 (17) | 133 (20) | 63 (27) |
| **Laboratory values 12h before until 6h after ICU admission** | | | | | | | |
| First lactate, mmol/L | 1.7 [1.1-3.4] | 1.0 [0.7-1.2] | 1.7 [1.4-2.1] | 2.6 [2.1-3.4] | 4.3 [3.4-5.5] | 7.2 [5.6-9.3] | 13.9 [11.1-17.0] |
| Mean lactate, mmol/L | 1.7 [1.1-3.0] | 1.0 [0.8-1.1] | 1.6 [1.5-1.8] | 2.5 [2.3-2.8] | 4.0 [3.6-4.4] | 6.5 [5.7-7.8] | 13.4 [11.1-17.0] |
| Maximum lactate, mmol/L | 2.3 [1.4-4.2] | 1.2 [0.9-1.5] | 2.1 [1.8-2.5] | 3.3 [2.8-4.2] | 5.2 [4.4-6.5] | 8.7 [7.1-10.5] | 16.0 [13.2-19.4] |
| No. of lactate measurements | 4 [2-6] | 4 [2-6] | 4 [2-6] | 5 [2-7] | 4 [2-6] | 4 [2-7] | 5 [2-8] |
| Mean glucose, mmol/L[g] | 10.3 [8.0-13.6] | 8.8 [7.2-10.9] | 10.4 [8.5-13.0] | 11.9 [9.1-15.5] | 12.8 [9.7-16.6] | 12.9 [9.3-17.6] | 11.2 [8.2-16.1] |

**Additional file 1 -** Prognostic impact of elevated lactate levels on mortality in critically ill patients with and without preadmission metformin treatment: a Danish registry-based cohort study

| | | | | | | | |
|---|---|---|---|---|---|---|---|
| **ICU treatment** | | | | | | | |
| Mechanical ventilation | 3150 (41) | 989 (35) | 711 (40) | 590 (42) | 360 (44) | 342 (51) | 158 (67) |
| Inotropes or vasopressors | 3007 (39) | 809 (29) | 635 (36) | 617 (44) | 397 (49) | 384 (57) | 165 (70) |
| Renal replacement therapy | 581 (8) | 144 (5) | 94 (5) | 100 (7) | 69 (8) | 95 (14) | 79 (33) |
| ICU length of stay, days[h] | 1.2 [0.7-2.9] | 1.1 [0.8-2.5] | 1.1 [0.8-2.6] | 1.5 [0.7-3.5] | 1.7 [0.8-3.9] | 1.3 [0.6-3.6] | 1.5 [0.6-4.6] |
| Hospital length of stay, days | 11 [6-20] | 11 [6-20] | 11 [6-21] | 11 [5-20] | 11 [5-22] | 10 [4-21] | 8 [2-17] |
| **Outcome** | | | | | | | |
| 30-day mortality | 1788 (23) | 430 (15) | 341 (19) | 353 (25) | 244 (30) | 279 (41) | 141 (59) |
| 90-day mortality | 2241 (29) | 596 (21) | 430 (24) | 443 (32) | 299 (37) | 323 (48) | 150 (63) |

Blood lactate level category based on quintiles and clinically relevant boundaries in mmol/L. Data are expressed as n (%) or median [IQR].

[a] Based on international classification of diseases codes only.

[b] According to Danish regulation, frequencies smaller than five patients not being zero and the corresponding total number of patients are not reported.

[c] Data missing for 440 (6%) patients.

[d] Data missing for 5132 (67%) patients.

[e] Data missing for 394 (5%) patients.

[f] Data missing for 5130 (67%) patients.

[g] Data missing for 152 (2%) patients.

[h] Data missing for 496 (6%) patients.

**Additional file 1 -** Prognostic impact of elevated lactate levels on mortality in critically ill patients with and without preadmission metformin treatment: a Danish registry-based cohort study

**Table S8 – Characteristics of metformin nonusers and users, when only including patients with diabetes**

| **Characteristic** | **Total** | **Metformin nonusers** | **Metformin users** |
|---|---|---|---|
| No. of patients | 7703 | 4524 | 3179 |
| Age, years | 70 [62-78] | 70 [60-78] | 70 [63-76] |
| Male | 4637 (60) | 2680 (59) | 1957 (62) |
| **Charlson comorbidity index score** | | | |
| 0 | 915 (12) | 388 (9) | 527 (17) |
| 1-2 | 2904 (38) | 1618 (36) | 1286 (40) |
| ≥3 | 3884 (50) | 2518 (56) | 1366 (43) |
| **Preadmission disease**[a] | | | |
| Myocardial infarction | 875 (11) | 579 (13) | 296 (9) |
| Congestive heart failure | 1364 (18) | 894 (20) | 470 (15) |
| Peripheral vascular disease | 1309 (17) | 843 (19) | 466 (15) |
| Cerebrovascular disease | 1360 (18) | 836 (18) | 524 (16) |
| Dementia | 140 (2) | 80 (2) | 60 (2) |
| Chronic pulmonary disease | 1728 (22) | 1082 (24) | 646 (20) |
| Connective tissue disease | 400 (5) | 263 (6) | 137 (4) |
| Ulcer disease | 553 (7) | 369 (8) | 184 (6) |
| Mild liver disease | 314 (4) | 228 (5) | 86 (3) |
| Moderate to severe liver disease | 147 (2) | 109 (2) | 38 (1) |
| Diabetes without end-organ failure | 4855 (63) | 2993 (66) | 1862 (59) |
| Diabetes with end-organ failure | 2698 (35) | 1804 (40) | 894 (28) |
| Moderate to severe renal disease | 967 (13) | 801 (18) | 166 (5) |
| Hemiplegia | 49 (1) | 28 (1) | 21 (1) |
| Any tumor | 1244 (16) | 727 (16) | 517 (16) |
| Metastatic solid tumor | 250 (3) | 166 (4) | 84 (3) |
| Leukemia | 51 (1) | 35 (1) | 16 (1) |
| Lymphoma | 127 (2) | 88 (2) | 39 (1) |
| AIDS[b] | <10 | 5 (0) | <5 |
| **Renal function during 1 year before ICU admission**[c] | | | |
| eGFR, ml/min/1.73m$^2$ | 71 [48-91] | 66 [41-90] | 77 [58-92] |
| ≥60 ml/min/1.73m$^2$ | 4568 (63) | 2339 (55) | 2229 (73) |
| 45-60 ml/min/1.73m$^2$ | 1138 (16) | 654 (15) | 484 (16) |
| 30-45 ml/min/1.73m$^2$ | 930 (13) | 670 (16) | 260 (9) |
| <30 ml/min/1.73m$^2$ | 627 (9) | 563 (13) | 64 (2) |
| Dialysis before ICU admission[b] | <137 | 132 (3) | <5 |
| **Antihyperglycemic therapy** | | | |
| Sulfonylureas | 899 (12) | 410 (9) | 489 (15) |
| Insulin | 2343 (30) | 1652 (37) | 691 (22) |
| Other antihyperglycemic agents | 691 (9) | 281 (6) | 410 (13) |
| HbA1c within 4 weeks before ICU admission, %[d] | 6.9 [6.4-8.0] | 6.9 [6.4-8.2] | 6.9 [6.3-7.8] |
| **ICU admission type**[e] | | | |
| Medical | 4186 (57) | 2550 (59) | 1636 (54) |
| Emergency surgery | 1822 (25) | 1113 (26) | 709 (24) |

**Additional file 1 -** Prognostic impact of elevated lactate levels on mortality in critically ill patients with and without preadmission metformin treatment: a Danish registry-based cohort study

| | | | |
|---|---|---|---|
| Elective surgery | 1301 (18) | 630 (15) | 671 (22) |
| SAPS-II score[f] | 42 [31-54] | 42 [31-53] | 42 [31-54] |
| **Primary diagnostic category** | | | |
| Cardiovascular | 1829 (24) | 993 (22) | 836 (26) |
| Respiratory, incl. pneumonia | 1222 (16) | 699 (15) | 523 (16) |
| Infection or sepsis, excl. pneumonia | 836 (11) | 534 (12) | 302 (9) |
| Gastrointestinal and liver disease | 808 (10) | 499 (11) | 309 (10) |
| Neoplasms | 667 (9) | 360 (8) | 307 (10) |
| Trauma and poisoning | 622 (8) | 357 (8) | 265 (8) |
| Endocrinology | 583 (8) | 442 (10) | 141 (4) |
| Other | 1136 (15) | 640 (14) | 496 (16) |
| **Laboratory values 12h before until 6h after ICU admission** | | | |
| First lactate, mmol/L | 1.7 [1.1-3.4] | 1.7 [1.0-3.2] | 1.8 [1.1-3.7] |
| Mean lactate, mmol/L | 1.7 [1.1-3.0] | 1.6 [1.1-2.9] | 1.8 [1.2-3.2] |
| Maximum lactate, mmol/L | 2.3 [1.4-4.2] | 2.2 [1.3-4.0] | 2.4 [1.5-4.5] |
| No. of lactate measurements | 4 [2-6] | 4 [2-6] | 4 [2-6] |
| Mean glucose, mmol/L[g] | 10.3 [8.0-13.6] | 10.3 [7.9-13.9] | 10.2 [8.2-13.1] |
| **ICU treatment** | | | |
| Mechanical ventilation | 3150 (41) | 1761 (39) | 1389 (44) |
| Inotropes or vasopressors | 3007 (39) | 1750 (39) | 1257 (40) |
| Renal replacement therapy | 581 (8) | 359 (8) | 222 (7) |
| ICU length of stay, days[h] | 1.2 [0.7-2.9] | 1.2 [0.7-2.8] | 1.3 [0.8-3.1] |
| Hospital length of stay, days | 11 [6-20] | 11 [5-21] | 11 [6-20] |
| **Outcome** | | | |
| 30-day mortality | 1788 (23) | 1170 (26) | 618 (19) |
| 90-day mortality | 2241 (29) | 1463 (32) | 778 (24) |

Data are expressed as n (%) or median [IQR].
[a] Based on international classification of diseases codes only.
[b] According to Danish regulation, frequencies smaller than five patients not being zero and the corresponding total number of patients are not reported.
[c] Data missing for 440 (6%) patients.
[d] Data missing for 5132 (67%) patients.
[e] Data missing for 394 (5%) patients.
[f] Data missing for 5130 (67%) patients.
[g] Data missing for 152 (2%) patients.
[h] Data missing for 496 (6%) patients.

**Additional file 1 -** Prognostic impact of elevated lactate levels on mortality in critically ill patients with and without preadmission metformin treatment: a Danish registry-based cohort study

**Table S9 – Thirty-day mortality, hazard ratios, and the relative excess risk due to interaction by lactate category for metformin nonusers and users among patients with diabetes mellitus**

| | **Events / no. at risk** | | **Crude HR (95% CI)** | | **Adjusted HR (95% CI)[a]** | | **RERI (95% CI)[b]** |
|---|---|---|---|---|---|---|---|
| Lactate (mmol/L) | Metformin nonusers | Metformin users | Metformin nonusers | Metformin users | Metformin nonusers | Metformin users | |
| <1.4 | 294 / 1743 (17%) | 136 / 1047 (13%) | Reference | 0.75 (0.61 to 0.92) | Reference | 0.82 (0.67 to 1.01) | |
| 1.4 to 2.0 | 227 / 1031 (22%) | 114 / 756 (15%) | 1.36 (1.14 to 1.62) | 0.89 (0.71 to 1.10) | 1.42 (1.19 to 1.69) | 0.99 (0.79 to 1.23) | -0.26 (-0.58 to 0.07) |
| 2.0 to 3.2 | 237 / 809 (29%) | 116 / 595 (19%) | 1.89 (1.60 to 2.25) | 1.18 (0.95 to 1.47) | 2.07 (1.74 to 2.47) | 1.33 (1.07 to 1.66) | -0.56 (-0.99 to -0.14) |
| 3.2 to 5.0 | 160 / 460 (35%) | 84 / 352 (24%) | 2.35 (1.94 to 2.85) | 1.47 (1.15 to 1.87) | 2.51 (2.07 to 3.05) | 1.63 (1.27 to 2.08) | -0.70 (-1.28 to -0.13) |
| 5.0 to 10.0 | 174 / 373 (47%) | 105 / 300 (35%) | 3.66 (3.03 to 4.42) | 2.44 (1.95 to 3.05) | 4.30 (3.55 to 5.20) | 2.72 (2.17 to 3.41) | -1.40 (-2.29 to -0.51) |
| ≥10.0 | 78 / 108 (72%) | 63 / 129 (49%) | 7.51 (5.85 to 9.64) | 4.03 (3.07 to 5.29) | 8.02 (6.22 to 10.35) | 4.83 (3.66 to 6.38) | -3.01 (-5.24 to -0.78) |

Hazard ratio (HR) with 95% confidence interval (95% CI) was computed using Cox proportional regression analysis based on muliple imputed dataset including only patients with diabetes mellitus.

[a] Adjusted for age, gender, last HbA1c measurement within 4 weeks before ICU admission, mean of all creatinine measurements 1 year before ICU admission, concomitant use of insulin, sulfonylurea derivates, or other antihyperglycemic drugs 90 days before ICU admission, and previous diagnosis of myocardial infarction, congestive heart failure, peripheral artery disease, cerebrovascular disease, dementia, connective tissue disease, peptic ulcer disease, chronic pulmonary disease, mild to severe liver disease, any tumor, metastatic solid tumor, leukemia, and lymphoma, respectively, 10 years before ICU admission. Codes used for each variable are stated in Appendix 1.

[b] Relative excess risk due to interaction (RERI) quantifies interaction on an additive scale, which approaches zero in the absence of interaction.

**Additional file 1 -** Prognostic impact of elevated lactate levels on mortality in critically ill patients with and without preadmission metformin treatment: a Danish registry-based cohort study

**Table S10 – Characteristics of patients that underwent elective surgery stratified by metformin use and stratified by lactate level category**

| **Characteristic** | **Total** | **Metformin nonusers** | **Metformin users** | **Lactate <2.0 mmol/L** | **Lactate ≥2.0 mmol/L** |
|---|---|---|---|---|---|
| No. of patients | 6193 | 5521 | 672 | 5199 | 994 |
| Age, years | 69 [61-76] | 69 [60-76] | 70 [64-75] | 69 [61-76] | 67 [58-74] |
| Male | 3838 (62) | 3386 (61) | 452 (67) | 3278 (63) | 560 (56) |
| **Charlson comorbidity index score** | | | | | |
| 0 | 1893 (31) | 1804 (33) | 89 (13) | 1627 (31) | 266 (27) |
| 1-2 | 2604 (42) | 2306 (42) | 298 (44) | 2234 (43) | 370 (37) |
| ≥3 | 1696 (27) | 1411 (26) | 285 (42) | 1338 (26) | 358 (36) |
| **Preadmission disease[a]** | | | | | |
| Myocardial infarction | 656 (11) | 561 (10) | 95 (14) | 599 (12) | 57 (6) |
| Congestive heart failure | 518 (8) | 438 (8) | 80 (12) | 454 (9) | 64 (6) |
| Peripheral vascular disease | 816 (13) | 721 (13) | 95 (14) | 686 (13) | 130 (13) |
| Cerebrovascular disease | 795 (13) | 692 (13) | 103 (15) | 692 (13) | 103 (10) |
| Dementia | 43 (1) | 34 (1) | 9 (1) | 37 (1) | 6 (1) |
| Chronic pulmonary disease | 612 (10) | 539 (10) | 73 (11) | 519 (10) | 93 (9) |
| Connective tissue disease | 250 (4) | 226 (4) | 24 (4) | 212 (4) | 38 (4) |
| Ulcer disease | 270 (4) | 235 (4) | 35 (5) | 232 (4) | 38 (4) |
| Mild liver disease | 85 (1) | 78 (1) | 7 (1) | 63 (1) | 22 (2) |
| Moderate to severe liver disease[b] | <25 (0) | 21 (0) | <5 | 16 (0) | 7 (1) |
| Diabetes without end-organ failure | 811 (13) | 391 (7) | 420 (62) | 684 (13) | 127 (13) |
| Diabetes with end-organ failure | 366 (6) | 204 (4) | 162 (24) | 314 (6) | 52 (5) |
| Moderate to severe renal disease | 258 (4) | 236 (4) | 22 (3) | 209 (4) | 49 (5) |
| Hemiplegia | 18 (0) | 18 (0) | 0 (0) | 10 (0) | 8 (1) |
| Any tumor | 1850 (30) | 1670 (30) | 180 (27) | 1429 (27) | 421 (42) |
| Metastatic solid tumor | 480 (8) | 456 (8) | 24 (4) | 332 (6) | 148 (15) |
| Leukemia[b] | <30 (0) | 24 (0) | <5 | 23 (0) | 3 (0) |
| Lymphoma | 70 (1) | 64 (1) | 6 (1) | 57 (1) | 13 (1) |
| AIDS[b] | <15 | 6 (0) | 0 (0) | <5 | <5 |
| **Renal function during 1 year before ICU admission[c]** | | | | | |
| eGFR, ml/min/1.73m$^2$ | 80 [63-92] | 80 [64-92] | 77 [61-91] | 80 [63-92] | 84 [67-95] |
| ≥60 ml/min/1.73m$^2$ | 4767 (80) | 4256 (80) | 511 (77) | 3989 (79) | 778 (82) |
| 45-60 ml/min/1.73m$^2$ | 697 (12) | 599 (11) | 98 (15) | 595 (12) | 102 (11) |
| 30-45 ml/min/1.73m$^2$ | 352 (6) | 307 (6) | 45 (7) | 303 (6) | 49 (5) |
| <30 ml/min/1.73m$^2$ | 164 (3) | 157 (3) | 7 (1) | 148 (3) | 16 (2) |
| Dialysis before ICU admission | 33 (1) | 33 (1) | 0 (0) | 28 (1) | 5 (1) |
| **Antihyperglycemic therapy** | | | | | |
| Metformin | 672 (11) | 0 (0) | 672 (100) | 546 (11) | 126 (13) |
| Sulfonylureas | 182 (3) | 74 (1) | 108 (16) | 152 (3) | 30 (3) |
| Insulin | 352 (6) | 206 (4) | 146 (22) | 296 (6) | 56 (6) |
| Other antihyperglycemic agents | 126 (2) | 46 (1) | 80 (12) | 102 (2) | 24 (2) |

**Additional file 1 -** Prognostic impact of elevated lactate levels on mortality in critically ill patients with and without preadmission metformin treatment: a Danish registry-based cohort study

| | | | | | |
|---|---|---|---|---|---|
| HbA1c within 4 weeks before ICU admission, %[d] | 5.8 [5.5-6.4] | 5.7 [5.4-6.1] | 6.9 [6.4-7.7] | 5.8 [5.4-6.4] | 6.0 [5.5-6.9] |
| SAPS-II score[e] | 29 [20-37] | 29 [20-37] | 28 [19.5-36] | 27 [20-34.8] | 35 [24-46] |
| **Primary diagnostic category** | | | | | |
| Cardiovascular | 3093 (50) | 2708 (49) | 385 (57) | 2832 (54) | 261 (26) |
| Infection or sepsis | 117 (2) | 107 (2) | 10 (1) | 90 (2) | 27 (3) |
| Gastrointestinal and liver disease | 246 (4) | 223 (4) | 23 (3) | 175 (3) | 71 (7) |
| Neoplasms | 2050 (33) | 1870 (34) | 180 (27) | 1587 (31) | 463 (47) |
| Trauma and poisoning | 138 (2) | 130 (2) | 8 (1) | 98 (2) | 40 (4) |
| Other | 549 (9) | 483 (9) | 66 (10) | 417 (8) | 132 (13) |
| **Laboratory values 12h before until 6h after ICU admission** | | | | | |
| First lactate, mmol/L | 1.0 [0.7-1.4] | 1.0 [0.7-1.4] | 1.2 [0.9-1.6] | 0.9 [0.7-1.2] | 2.2 [1.5-3.0] |
| Mean lactate, mmol/L | 1.2 [0.9-1.7] | 1.2 [0.9-1.7] | 1.4 [1.0-1.8] | 1.1 [0.8-1.4] | 2.6 [2.3-3.3] |
| Maximum lactate, mmol/L | 1.6 [1.1-2.3] | 1.6 [1.0-2.3] | 1.8 [1.3-2.4] | 1.4 [1.0-1.9] | 3.6 [2.9-4.8] |
| No. of lactate measurements | 4 [2-6] | 4 [2-6] | 5 [3-7] | 4 [2-6] | 5 [2-7] |
| Mean glucose, mmol/L[f] | 7.7 [6.7-9.2] | 7.6 [6.6-8.9] | 9.3 [8.0-11.1] | 7.5 [6.6-8.7] | 9.4 [8.0-11.1] |
| **ICU treatment** | | | | | |
| Mechanical ventilation | 3004 (49) | 2628 (48) | 376 (56) | 2643 (51) | 361 (36) |
| Inotropes or vasopressors | 2080 (34) | 1836 (33) | 244 (36) | 1640 (32) | 440 (44) |
| Renal replacement therapy | 173 (3) | 157 (3) | 16 (2) | 102 (2) | 71 (7) |
| ICU length of stay, days[g] | 0.9 [0.9-1.3] | 0.9 [0.8-1.3] | 0.9 [0.9-1.8] | 0.9 [0.9-1.1] | 1.0 [0.8-2.1] |
| Hospital length of stay, days | 11 [7-19] | 11 [7-19] | 11 [7-19] | 11 [7-18] | 12 [7-21] |
| **Outcome** | | | | | |
| 30-day mortality | 288 (5) | 260 (5) | 28 (4) | 173 (3) | 115 (12) |
| 90-day mortality | 424 (7) | 382 (7) | 42 (6) | 275 (5) | 149 (15) |

Data are expressed as n (%) or median [IQR].
[a] Based on international classification of diseases codes only.
[b] According to Danish regulation, frequencies smaller than five patients not being zero and the corresponding total number of patients are not reported.
[c] Data missing for 213 (3%) patients.
[d] Data missing for 4755 (77%) patients.
[e] Data missing for 5177 (84%) patients.
[f] Data missing for 134 (2%) patients.
[h] Data missing for 186 (3%) patients.

**Additional file 1 -** Prognostic impact of elevated lactate levels on mortality in critically ill patients with and without preadmission metformin treatment: a Danish registry-based cohort study

**Table S11 – Thirty-day mortality, hazard ratios, and the relative excess risk due to interaction by lactate category for metformin nonusers and users undergoing elective surgery**

| | **Events / no. at risk** | | **Crude HR (95% CI)** | | **Adjusted HR (95% CI)[a]** | | **RERI (95% CI)[b]** |
|---|---|---|---|---|---|---|---|
| Lactate (mmol/L) | Metformin nonusers | Metformin users | Metformin nonusers | Metformin users | Metformin nonusers | Metformin users | |
| <2.0 | 159 / 4653 (3%) | 14 / 546 (3%) | Reference | 0.75 (0.43 to 1.29) | Reference | 0.61 (0.34 to 1.09) | |
| ≥2.0 | 101 / 868 (12%) | 14 / 126 (11%) | 3.61 (2.81 to 4.63) | 3.43 (1.98 to 5.92) | 4.17 (3.23 to 5.38) | 3.14 (1.72 to 5.75) | -0.64 (-2.65 to 1.38) |

Hazard ratio (HR) with 95% confidence interval (95% CI) was computed using Cox proportional regression analysis based on muliple imputed dataset including patients undergoing elective surgery.

[a] Adjusted for age, gender, last HbA1c measurement within 4 weeks before ICU admission, mean of all creatinine measurements 1 year before ICU admission, and Charlson Comorbidity Index. Codes used for each variable are stated in Appendix 1.

[b] Relative excess risk due to interaction (RERI) quantifies interaction on an additive scale, which approaches zero in the absence of interaction.

**Additional file 1 -** Prognostic impact of elevated lactate levels on mortality in critically ill patients with and without preadmission metformin treatment: a Danish registry-based cohort study

**Table S12 – Characteristics of metformin nonusers and metformin users, stratified by preadmission estimated glomerular filtration rate (eGFR)**

| | | **eGFR ≥60 ml/min/1.73m²** | | **eGFR <60 ml/min/1.73m²** | |
|---|---|---|---|---|---|
| **Characteristic** | **Total** | **Metformin nonusers** | **Metformin users** | **Metformin nonusers** | **Metformin users** |
| No. of patients | 30,586 | 20,539 | 2231 | 7007 | 809 |
| Age, years | 69 [59-78] | 66 [55-74] | 68 [62-74] | 78 [71-83] | 75 [69-80] |
| Male | 17,243 (56) | 11,605 (57) | 1432 (64) | 3758 (54) | 448 (55) |
| **Charlson comorbidity index score** | | | | | |
| 0 | 9098 (30) | 7418 (36) | 398 (18) | 1184 (17) | 98 (12) |
| 1-2 | 12,489 (41) | 8759 (43) | 961 (43) | 2501 (36) | 268 (33) |
| ≥3 | 8999 (29) | 4362 (21) | 872 (39) | 3322 (47) | 443 (55) |
| **Preadmission disease**[a] | | | | | |
| Myocardial infarction | 2460 (8) | 1263 (6) | 178 (8) | 910 (13) | 109 (13) |
| Congestive heart failure | 3324 (11) | 1317 (6) | 257 (12) | 1557 (22) | 193 (24) |
| Peripheral vascular disease | 3826 (13) | 2026 (10) | 292 (13) | 1349 (19) | 159 (20) |
| Cerebrovascular disease | 4615 (15) | 2694 (13) | 352 (16) | 1418 (20) | 151 (19) |
| Dementia | 505 (2) | 271 (1) | 41 (2) | 180 (3) | 13 (2) |
| Chronic pulmonary disease | 6226 (20) | 3922 (19) | 433 (19) | 1683 (24) | 188 (23) |
| Connective tissue disease | 1589 (5) | 969 (5) | 86 (4) | 489 (7) | 45 (6) |
| Ulcer disease | 1980 (6) | 1174 (6) | 117 (5) | 628 (9) | 61 (8) |
| Mild liver disease | 1208 (4) | 915 (4) | 68 (3) | 209 (3) | 16 (2) |
| Moderate to severe liver disease | 514 (2) | 380 (2) | 29 (1) | 99 (1) | 6 (1) |
| Diabetes without end-organ failure | 4571 (15) | 1480 (7) | 1281 (57) | 1300 (19) | 510 (63) |
| Diabetes with end-organ failure | 2557 (8) | 695 (3) | 563 (25) | 1002 (14) | 297 (37) |
| Moderate to severe renal disease | 2324 (8) | 297 (1) | 56 (3) | 1871 (27) | 100 (12) |
| Hemiplegia[b] | <300 (1) | 242 (1) | 17 (1) | 33 (0) | <5 |
| Any tumor | 5641 (18) | 3773 (18) | 368 (16) | 1366 (19) | 134 (17) |
| Metastatic solid tumor | 1362 (4) | 1022 (5) | 66 (3) | 256 (4) | 18 (2) |
| Leukemia | 275 (1) | 179 (1) | 12 (1) | 80 (1) | 4 (0) |
| Lymphoma | 623 (2) | 395 (2) | 24 (1) | 191 (3) | 13 (2) |
| AIDS[b] | <50 (0) | 30 (0) | <5 | 10 (0) | <5 |
| **Renal function during 1 year before ICU admission** | | | | | |
| eGFR, ml/min/1.73m² | 80 [59-95] | 89 [76-100] | 86 [73-96] | 43 [31-52] | 47 [40-54] |
| ≥60 ml/min/1.73m² | 22,770 (74) | 20,539 (100) | 2231 (100) | 0 (0) | 0 (0) |
| 45-60 ml/min/1.73m² | 3773 (12) | 0 (0) | 0 (0) | 3289 (47) | 484 (60) |
| 30-45 ml/min/1.73m² | 2402 (8) | 0 (0) | 0 (0) | 2142 (31) | 260 (32) |
| <30 ml/min/1.73m² | 1641 (5) | 0 (0) | 0 (0) | 1576 (22) | 65 (8) |
| Dialysis before ICU admission[b] | <320 (1) | 0 (0) | 0 (0) | 315 (4) | <5 |
| **Antihyperglycemic therapy** | | | | | |
| Sulfonylureas | 841 (3) | 162 (1) | 330 (15) | 220 (3) | 129 (16) |
| Insulin | 2204 (7) | 755 (4) | 453 (20) | 778 (11) | 218 (27) |
| Other antihyperglycemic agents | 672 (2) | 106 (1) | 274 (12) | 166 (2) | 126 (16) |

**Additional file 1 -** Prognostic impact of elevated lactate levels on mortality in critically ill patients with and without preadmission metformin treatment: a Danish registry-based cohort study

| | | | | | |
|---|---|---|---|---|---|
| HbA1c within 4 weeks before ICU admission, %[c] | 6.0 [5.5-6.7] | 5.7 [5.4-6.2] | 6.9 [6.3-7.8] | 6.1 [5.6-6.8] | 6.9 [6.3-7.7] |
| **ICU admission type[d]** | | | | | |
| Medical | 14,412 (50) | 9334 (49) | 1109 (52) | 3532 (53) | 437 (57) |
| Emergency surgery | 8314 (29) | 5564 (29) | 499 (24) | 2073 (31) | 178 (23) |
| Elective surgery | 5980 (21) | 4256 (22) | 511 (24) | 1063 (16) | 150 (20) |
| SAPS-II score[e] | 40 [30-53] | 38 [29-50] | 41 [29-53] | 45 [35-56] | 44 [35-54] |
| **Primary diagnostic category** | | | | | |
| Cardiovascular | 7054 (23) | 4630 (23) | 586 (26) | 1621 (23) | 217 (27) |
| Respiratory, incl. pneumonia | 4451 (15) | 2870 (14) | 364 (16) | 1087 (16) | 130 (16) |
| Infection or sepsis, excl. pneumonia | 2841 (9) | 1685 (8) | 198 (9) | 873 (12) | 85 (11) |
| Gastrointestinal and liver disease | 3628 (12) | 2342 (11) | 208 (9) | 985 (14) | 93 (11) |
| Neoplasms | 3715 (12) | 2810 (14) | 246 (11) | 604 (9) | 55 (7) |
| Trauma and poisoning | 3304 (11) | 2513 (12) | 196 (9) | 541 (8) | 54 (7) |
| Endocrinology | 837 (3) | 521 (3) | 96 (4) | 186 (3) | 34 (4) |
| Other | 4756 (16) | 3168 (15) | 337 (15) | 1110 (16) | 141 (17) |
| **Laboratory values 12h before until 6h after ICU admission** | | | | | |
| First lactate, mmol/L | 1.5 [1.0-2.9] | 1.5 [0.9-2.9] | 1.8 [1.1-3.7] | 1.5 [0.9-2.9] | 1.8 [1.1-3.7] |
| Mean lactate, mmol/L | 1.6 [1.0-2.7] | 1.6 [1.0-2.7] | 1.8 [1.2-3.2] | 1.5 [1.0-2.6] | 1.8 [1.1-3.2] |
| Maximum lactate, mmol/L | 2.1 [1.3-3.7] | 2.0 [1.3-3.6] | 2.3 [1.5-4.5] | 2.0 [1.2-3.6] | 2.4 [1.4-4.6] |
| No. of lactate measurements | 4 [2-6] | 4 [2-6] | 4 [2-6] | 4 [2-6] | 4 [2-6] |
| Mean glucose, mmol/L[f] | 8.1 [6.7-10.2] | 7.9 [6.6-9.6] | 10.2 [8.2-13.1] | 8.2 [6.7-10.3] | 10.2 [8.1-12.9] |
| **ICU treatment** | | | | | |
| Mechanical ventilation | 12,626 (41) | 8621 (42) | 996 (45) | 2683 (38) | 326 (40) |
| Inotropes or vasopressors | 11,056 (36) | 6962 (34) | 858 (38) | 2891 (41) | 345 (43) |
| Renal replacement therapy | 1666 (5) | 746 (4) | 131 (6) | 704 (10) | 85 (11) |
| ICU length of stay, days[g] | 1.1 [0.7-2.8] | 1.0 [0.7-2.7] | 1.2 [0.8-3.0] | 1.2 [0.7-3.0] | 1.6 [0.8-3.4] |
| Hospital length of stay, days | 10 [5-21] | 10 [5-20] | 11 [6-19] | 11 [6-21] | 12 [6-21] |
| **Outcome** | | | | | |
| 30-day mortality | 6668 (22) | 3845 (19) | 377 (17) | 2243 (32) | 203 (25) |
| 90-day mortality | 8248 (27) | 4738 (23) | 489 (22) | 2779 (40) | 242 (30) |

Data are expressed as n (%) or median [IQR].
[a] Based on international classification of diseases codes only.
[b] According to Danish regulation, frequencies smaller than five patients not being zero and the corresponding total number of patients are not reported.
[c] Data missing for 25,119 (82%) patients.
[d] Data missing for 1880 (6%) patients.
[e] Data missing for 21,153 (69%) patients
[f] Data missing for 663 (2%) patients.
[g] Data missing for 2188 (7%) patients.

**Table S13 – Characteristics of patients with preadmission estimated glomerular filtration rate (eGFR) ≥60 ml/min/1.72m$^2$, stratified by lactate level category**

| Characteristic | Total | <1.4 | 1.4 to 2.0 | 2.0 to 3.2 | 3.2 to 5.0 | 5.0 to 10.0 | ≥10 |
|---|---|---|---|---|---|---|---|
| No. of patients | 22,770 | 9029 | 5399 | 4041 | 2234 | 1506 | 561 |
| Age, years | 66 [55-74] | 67 [57-74] | 66 [55-74] | 66 [54-74] | 66 [55-74] | 66 [55-75] | 63 [54-71] |
| Male | 13,037 (57) | 5014 (56) | 3089 (57) | 2355 (58) | 1302 (58) | 926 (61) | 351 (63) |
| **Charlson comorbidity index score** | | | | | | | |
| 0 | 7816 (34) | 3084 (34) | 1901 (35) | 1390 (34) | 730 (33) | 512 (34) | 199 (35) |
| 1-2 | 9720 (43) | 3980 (44) | 2289 (42) | 1685 (42) | 939 (42) | 611 (41) | 216 (39) |
| ≥3 | 5234 (23) | 1965 (22) | 1209 (22) | 966 (24) | 565 (25) | 383 (25) | 146 (26) |
| **Preadmission disease**[a] | | | | | | | |
| Myocardial infarction | 1441 (6) | 623 (7) | 360 (7) | 208 (5) | 137 (6) | 87 (6) | 26 (5) |
| Congestive heart failure | 1574 (7) | 611 (7) | 399 (7) | 258 (6) | 155 (7) | 111 (7) | 40 (7) |
| Peripheral vascular disease | 2318 (10) | 937 (10) | 593 (11) | 375 (9) | 212 (9) | 154 (10) | 47 (8) |
| Cerebrovascular disease | 3046 (13) | 1249 (14) | 692 (13) | 516 (13) | 313 (14) | 197 (13) | 79 (14) |
| Dementia | 312 (1) | 106 (1) | 71 (1) | 60 (1) | 41 (2) | 24 (2) | 10 (2) |
| Chronic pulmonary disease | 4355 (19) | 1914 (21) | 1004 (19) | 727 (18) | 395 (18) | 243 (16) | 72 (13) |
| Connective tissue disease | 1055 (5) | 394 (4) | 261 (5) | 190 (5) | 112 (5) | 68 (5) | 30 (5) |
| Ulcer disease | 1291 (6) | 513 (6) | 263 (5) | 235 (6) | 131 (6) | 96 (6) | 53 (9) |
| Mild liver disease | 983 (4) | 253 (3) | 193 (4) | 182 (5) | 142 (6) | 135 (9) | 78 (14) |
| Moderate to severe liver disease | 409 (2) | 83 (1) | 81 (2) | 79 (2) | 63 (3) | 66 (4) | 37 (7) |
| Diabetes without end-organ failure | 2761 (12) | 972 (11) | 660 (12) | 504 (12) | 305 (14) | 235 (16) | 85 (15) |
| Diabetes with end-organ failure | 1258 (6) | 432 (5) | 290 (5) | 227 (6) | 137 (6) | 134 (9) | 38 (7) |
| Moderate to severe renal disease | 353 (2) | 127 (1) | 76 (1) | 74 (2) | 42 (2) | 28 (2) | 6 (1) |
| Hemiplegia | 259 (1) | 126 (1) | 59 (1) | 43 (1) | 16 (1) | 10 (1) | 5 (1) |
| Any tumor | 4141 (18) | 1655 (18) | 979 (18) | 779 (19) | 432 (19) | 222 (15) | 74 (13) |
| Metastatic solid tumor | 1088 (5) | 368 (4) | 258 (5) | 250 (6) | 139 (6) | 53 (4) | 20 (4) |
| Leukemia[b] | <195 (1) | 77 (1) | 42 (1) | 34 (1) | 18 (1) | 19 (1) | <5 |
| Lymphoma | 419 (2) | 154 (2) | 88 (2) | 78 (2) | 45 (2) | 49 (3) | 5 (1) |
| AIDS[b] | <40 | 11 (0) | 7 (0) | 9 (0) | <5 | 0 (0) | <5 |
| **Renal function during 1 year before ICU admission** | | | | | | | |
| eGFR, ml/min/1.73m$^2$ | 88 [76-99] | 88 [76-99] | 88 [76-99] | 89 [76-100] | 89 [76-100] | 89 [76-101] | 90 [79-103] |
| Dialysis before ICU admission | 0 (0) | 0 (0) | 0 (0) | 0 (0) | 0 (0) | 0 (0) | 0 (0) |
| **Antihyperglycemic therapy** | | | | | | | |
| Metformin | 2231 (10) | 732 (8) | 557 (10) | 398 (10) | 248 (11) | 211 (14) | 85 (15) |
| Sulfonylureas | 492 (2) | 152 (2) | 131 (2) | 84 (2) | 60 (3) | 42 (3) | 23 (4) |
| Insulin | 1208 (5) | 394 (4) | 302 (6) | 239 (6) | 128 (6) | 116 (8) | 29 (5) |
| Other antihyperglycemic agents | 380 (2) | 97 (1) | 102 (2) | 75 (2) | 49 (2) | 37 (2) | 20 (4) |

**Additional file 1 -** Prognostic impact of elevated lactate levels on mortality in critically ill patients with and without preadmission metformin treatment: a Danish registry-based cohort study

| | | | | | | | |
|---|---|---|---|---|---|---|---|
| HbA1c within 4 weeks before ICU admission, %[c] | 5.9 [5.5-6.6] | 5.8 [5.5-6.4] | 5.9 [5.4-6.7] | 6.1 [5.6-7.0] | 6.0 [5.4-6.9] | 6.0 [5.4-6.7] | 5.8 [5.4-7.0] |
| **ICU admission type[d]** | | | | | | | |
| Medical | 10,443 (49) | 3430 (41) | 2361 (47) | 2017 (54) | 1307 (63) | 944 (67) | 384 (73) |
| Emergency surgery | 6063 (29) | 2293 (27) | 1435 (28) | 1151 (31) | 620 (30) | 429 (30) | 135 (26) |
| Elective surgery | 4767 (22) | 2740 (32) | 1249 (25) | 579 (15) | 151 (7) | 42 (3) | 6 (1) |
| SAPS-II score[e] | 39 [29-51] | 33 [25-43] | 37 [27-47] | 40 [31-52] | 45 [35-59] | 53 [42-67] | 62 [46-73] |
| **Primary diagnostic category** | | | | | | | |
| Cardiovascular | 5216 (23) | 2371 (26) | 1223 (23) | 670 (17) | 420 (19) | 363 (24) | 169 (30) |
| Respiratory, incl. pneumonia | 3234 (14) | 1374 (15) | 776 (14) | 592 (15) | 311 (14) | 151 (10) | 30 (5) |
| Infection or sepsis, excl. pneumonia | 1883 (8) | 550 (6) | 441 (8) | 367 (9) | 276 (12) | 197 (13) | 52 (9) |
| Gastrointestinal and liver disease | 2550 (11) | 956 (11) | 562 (10) | 492 (12) | 267 (12) | 197 (13) | 76 (14) |
| Neoplasms | 3056 (13) | 1362 (15) | 776 (14) | 574 (14) | 221 (10) | 98 (7) | 25 (4) |
| Trauma and poisoning | 2709 (12) | 1038 (11) | 665 (12) | 569 (14) | 253 (11) | 146 (10) | 38 (7) |
| Endocrinology | 617 (3) | 227 (3) | 127 (2) | 134 (3) | 60 (3) | 54 (4) | 15 (3) |
| Other | 3505 (15) | 1151 (13) | 829 (15) | 643 (16) | 426 (19) | 300 (20) | 156 (28) |
| **Laboratory values 12h before until 6h after ICU admission** | | | | | | | |
| First lactate, mmol/L | 1.6 [1.0-2.9] | 0.9 [0.7-1.2] | 1.6 [1.3-2.0] | 2.6 [2.1-3.3] | 4.3 [3.4-5.6] | 7.4 [5.6-9.7] | 14.1 [11.5-17.0] |
| Mean lactate, mmol/L | 1.6 [1.0-2.7] | 0.9 [0.8-1.1] | 1.6 [1.5-1.8] | 2.5 [2.3-2.8] | 3.9 [3.5-4.4] | 6.5 [5.7-8.0] | 12.9 [11.2-15.9] |
| Maximum lactate, mmol/L | 2.1 [1.3-3.7] | 1.1 [0.9-1.4] | 2.1 [1.8-2.5] | 3.2 [2.7-4.0] | 5.1 [4.3-6.4] | 8.8 [7.2-10.9] | 15.0 [13.0-19.0] |
| No. of lactate measurements | 4 [2-6] | 3 [2-5] | 4 [2-6] | 4 [2-6] | 4 [2-6] | 4 [2-7] | 3 [1-7] |
| Mean glucose, mmol/L[f] | 8.0 [6.7-10.0] | 7.3 [6.3-8.6] | 8.3 [7.0-9.9] | 8.9 [7.3-10.9] | 9.5 [7.4-12.0] | 9.9 [7.2-13.5] | 9.6 [6.6-13.8] |
| **ICU treatment** | | | | | | | |
| Mechanical ventilation | 9617 (42) | 3467 (38) | 2156 (40) | 1668 (41) | 1106 (50) | 845 (56) | 375 (67) |
| Inotropes or vasopressors | 7820 (34) | 2267 (25) | 1725 (32) | 1543 (38) | 1079 (48) | 847 (56) | 359 (64) |
| Renal replacement therapy | 877 (4) | 143 (2) | 128 (2) | 164 (4) | 151 (7) | 183 (12) | 108 (19) |
| ICU length of stay, days[g] | 1.0 [0.7-2.7] | 1.0 [0.7-2.0] | 1.0 [0.7-2.5] | 1.2 [0.6-3.4] | 1.5 [0.7-4.3] | 1.4 [0.6-4.4] | 1.1 [0.5-3.4] |
| Hospital length of stay, days | 10 [5-20] | 10 [6-19] | 11 [6-21] | 11 [5-21] | 11 [5-22] | 9 [3-21] | 5 [2-14] |
| **Outcome** | | | | | | | |
| 30-day mortality | 4222 (19) | 1002 (11) | 820 (15) | 811 (20) | 657 (29) | 602 (40) | 330 (59) |
| 90-day mortality | 5227 (23) | 1372 (15) | 1052 (19) | 1010 (25) | 771 (35) | 673 (45) | 349 (62) |

Data are expressed as n (%) or median [IQR]. [a] Based on international classification of diseases codes only.
[b] According to Danish regulation, frequencies smaller than five patients not being zero and the corresponding total number of patients are not reported. [c] Data missing for 25,119 (82%) patients. [d] Data missing for 1497 (7%) patients. [e] Data missing for 16,059 (71%) patients. [f] Data missing for 492 (2%) patients. [g] Data missing for 1743 (8%) patients.

**Additional file 1 -** Prognostic impact of elevated lactate levels on mortality in critically ill patients with and without preadmission metformin treatment: a Danish registry-based cohort study

**Table S14 – Characteristics of patients with preadmission estimated glomerular filtration rate (eGFR) <60 ml/min/1.72m$^2$, stratified by lactate level category**

| Characteristic | Total | <1.4 | 1.4 to 2.0 | 2.0 to 3.2 | 3.2 to 5.0 | 5.0 to 10.0 | ≥10 |
|---|---|---|---|---|---|---|---|
| No. of patients | 7816 | 3306 | 1688 | 1355 | 776 | 523 | 168 |
| Age, years | 77 [70-83] | 77 [70-83] | 77 [70-83] | 78 [71-84] | 78 [70-84] | 77 [69-82] | 76 [69-81] |
| Male | 4206 (54) | 1777 (54) | 894 (53) | 747 (55) | 431 (56) | 270 (52) | 87 (52) |
| **Charlson comorbidity index score** | | | | | | | |
| 0 | 1282 (16) | 531 (16) | 272 (16) | 238 (18) | 119 (15) | 90 (17) | 32 (19) |
| 1-2 | 2769 (35) | 1157 (35) | 599 (35) | 490 (36) | 266 (34) | 200 (38) | 57 (34) |
| ≥3 | 3765 (48) | 1618 (49) | 817 (48) | 627 (46) | 391 (50) | 233 (45) | 79 (47) |
| **Preadmission disease[a]** | | | | | | | |
| Myocardial infarction | 1019 (13) | 421 (13) | 225 (13) | 188 (14) | 100 (13) | 59 (11) | 26 (15) |
| Congestive heart failure | 1750 (22) | 736 (22) | 398 (24) | 305 (23) | 167 (22) | 105 (20) | 39 (23) |
| Peripheral vascular disease | 1508 (19) | 617 (19) | 338 (20) | 259 (19) | 156 (20) | 100 (19) | 38 (23) |
| Cerebrovascular disease | 1569 (20) | 673 (20) | 346 (20) | 256 (19) | 160 (21) | 103 (20) | 31 (18) |
| Dementia | 193 (2) | 81 (2) | 40 (2) | 36 (3) | 23 (3) | 7 (1) | 6 (4) |
| Chronic pulmonary disease | 1871 (24) | 842 (25) | 395 (23) | 315 (23) | 192 (25) | 104 (20) | 23 (14) |
| Connective tissue disease | 534 (7) | 200 (6) | 128 (8) | 100 (7) | 56 (7) | 41 (8) | 9 (5) |
| Ulcer disease | 689 (9) | 302 (9) | 140 (8) | 122 (9) | 71 (9) | 44 (8) | 10 (6) |
| Mild liver disease | 225 (3) | 63 (2) | 51 (3) | 47 (3) | 27 (3) | 29 (6) | 8 (5) |
| Moderate to severe liver disease[b] | <110 (1) | 19 (1) | 28 (2) | 24 (2) | 17 (2) | 13 (2) | <5 (2) |
| Diabetes without end-organ failure | 1810 (23) | 740 (22) | 377 (22) | 305 (23) | 184 (24) | 154 (29) | 50 (30) |
| Diabetes with end-organ failure | 1299 (17) | 550 (17) | 277 (16) | 214 (16) | 127 (16) | 95 (18) | 36 (21) |
| Moderate to severe renal disease | 1971 (25) | 890 (27) | 415 (25) | 304 (22) | 205 (26) | 113 (22) | 44 (26) |
| Hemiplegia[b] | <45 (0) | 12 (0) | 8 (0) | 6 (0) | 5 (1) | <5 (1) | <5 (1) |
| Any tumor | 1500 (19) | 707 (21) | 308 (18) | 242 (18) | 141 (18) | 83 (16) | 19 (11) |
| Metastatic solid tumor[b] | <280 (4) | 109 (3) | 70 (4) | 40 (3) | 37 (5) | 15 (3) | <5 (2) |
| Leukemia[b] | <95 (1) | 30 (1) | 22 (1) | 16 (1) | 9 (1) | <5 (1) | <5 (2) |
| Lymphoma | 204 (3) | 76 (2) | 49 (3) | 31 (2) | 25 (3) | 16 (3) | 7 (4) |
| AIDS[b] | <25 (0) | <5 (0) | <5 (0) | 0 (0) | <5 (0) | <5 (0) | <5 (1) |
| **Renal function during 1 year before ICU admission** | | | | | | | |
| eGFR, ml/min/1.73m$^2$ | 44 [32-52] | 43 [31-52] | 44 [32-53] | 46 [34-53] | 44 [32-52] | 45 [34-53] | 45 [35-52] |
| 45-60 ml/min/1.73m$^2$ | 3773 (48) | 1531 (46) | 808 (48) | 713 (53) | 371 (48) | 263 (50) | 87 (52) |
| 30-45 ml/min/1.73m$^2$ | 2402 (31) | 1020 (31) | 528 (31) | 398 (29) | 242 (31) | 162 (31) | 52 (31) |
| <30 ml/min/1.73m$^2$ | 1641 (21) | 755 (23) | 352 (21) | 244 (18) | 163 (21) | 98 (19) | 29 (17) |
| Dialysis before ICU admission | 316 (4) | 124 (4) | 82 (5) | 47 (3) | 42 (5) | 15 (3) | 6 (4) |
| **Antihyperglycemic therapy** | | | | | | | |
| Metformin | 809 (10) | 277 (8) | 177 (10) | 159 (12) | 82 (11) | 76 (15) | 38 (23) |
| Sulfonylureas | 349 (4) | 139 (4) | 86 (5) | 54 (4) | 38 (5) | 26 (5) | 6 (4) |
| Insulin | 996 (13) | 418 (13) | 215 (13) | 154 (11) | 95 (12) | 94 (18) | 20 (12) |

**Additional file 1 -** Prognostic impact of elevated lactate levels on mortality in critically ill patients with and without preadmission metformin treatment: a Danish registry-based cohort study

| | | | | | | | |
|---|---|---|---|---|---|---|---|
| Other antihyperglycemic agents | 292 (4) | 105 (3) | 51 (3) | 52 (4) | 37 (5) | 32 (6) | 15 (9) |
| HbA1c within 4 weeks before ICU admission, %[c] | 6.2 [5.7-7.1] | 6.1 [5.6-6.9] | 6.2 [5.6-7.0] | 6.3 [5.7-7.2] | 6.2 [5.6-7.1] | 6.5 [5.9-7.5] | 6.4 [5.8-7.3] |
| **ICU admission type[d]** | | | | | | | |
| Medical | 3969 (53) | 1399 (44) | 820 (51) | 786 (61) | 504 (67) | 342 (69) | 118 (75) |
| Emergency surgery | 2251 (30) | 975 (31) | 502 (31) | 381 (30) | 213 (29) | 146 (29) | 34 (22) |
| Elective surgery | 1213 (16) | 772 (25) | 274 (17) | 122 (9) | 30 (4) | 10 (2) | 5 (3) |
| SAPS-II score[e] | 45 [35-56] | 40 [32-50] | 43 [35-53] | 47 [38-58] | 51 [43-65] | 58 [47-72] | 65 [54-81] |
| **Primary diagnostic category** | | | | | | | |
| Cardiovascular | 1838 (24) | 842 (25) | 391 (23) | 268 (20) | 155 (20) | 138 (26) | 44 (26) |
| Respiratory, incl. pneumonia | 1217 (16) | 523 (16) | 274 (16) | 231 (17) | 115 (15) | 57 (11) | 17 (10) |
| Infection or sepsis, excl. pneumonia | 958 (12) | 312 (9) | 210 (12) | 198 (15) | 143 (18) | 80 (15) | 15 (9) |
| Gastrointestinal and liver disease | 1078 (14) | 469 (14) | 235 (14) | 187 (14) | 99 (13) | 65 (12) | 23 (14) |
| Neoplasms | 659 (8) | 341 (10) | 151 (9) | 98 (7) | 45 (6) | 18 (3) | 6 (4) |
| Trauma and poisoning | 595 (8) | 280 (8) | 129 (8) | 107 (8) | 45 (6) | 26 (5) | 8 (5) |
| Endocrinology | 220 (3) | 82 (2) | 39 (2) | 53 (4) | 17 (2) | 20 (4) | 9 (5) |
| Other | 1251 (16) | 457 (14) | 259 (15) | 213 (16) | 157 (20) | 119 (23) | 46 (27) |
| **Laboratory values 12h before until 6h after ICU admission** | | | | | | | |
| First lactate, mmol/L | 1.5 [1.0-2.8] | 0.9 [0.7-1.2] | 1.6 [1.4-2.0] | 2.6 [2.1-3.4] | 4.1 [3.3-5.3] | 7.1 [5.6-9.1] | 12.9 [10.8-16.1] |
| Mean lactate, mmol/L | 1.5 [1.0-2.7] | 0.9 [0.7-1.1] | 1.6 [1.5-1.8] | 2.5 [2.3-2.8] | 4.0 [3.6-4.4] | 6.6 [5.7-7.8] | 12.9 [11.1-15.6] |
| Maximum lactate, mmol/L | 2.0 [1.2-3.7] | 1.1 [0.9-1.4] | 2.1 [1.8-2.5] | 3.3 [2.8-4.1] | 5.3 [4.4-6.4] | 8.6 [7.2-10.5] | 15.8 [12.9-19.0] |
| No. of lactate measurements | 4 [2-6] | 4 [2-6] | 4 [2-6] | 4 [2-6] | 4 [2-6] | 4 [2-7] | 4 [1-7] |
| Mean glucose, mmol/L[f] | 8.3 [6.8-10.6] | 7.5 [6.4-9.0] | 8.7 [7.2-10.6] | 9.4 [7.5-11.9] | 9.8 [7.6-13.0] | 10.5 [7.6-14.0] | 9.5 [7.1-13.5] |
| **ICU treatment** | | | | | | | |
| Mechanical ventilation | 3009 (38) | 1098 (33) | 609 (36) | 549 (41) | 359 (46) | 298 (57) | 96 (57) |
| Inotropes or vasopressors | 3236 (41) | 997 (30) | 702 (42) | 664 (49) | 432 (56) | 337 (64) | 104 (62) |
| Renal replacement therapy | 789 (10) | 258 (8) | 156 (9) | 134 (10) | 104 (13) | 96 (18) | 41 (24) |
| ICU length of stay, days[g] | 1.3 [0.7-3.0] | 1.1 [0.8-2.6] | 1.4 [0.8-3.1] | 1.7 [0.7-3.7] | 1.6 [0.7-3.8] | 1.5 [0.7-3.9] | 1.0 [0.4-3.1] |
| Hospital length of stay, days | 11 [6-21] | 11 [7-21] | 12 [7-23] | 11 [6-22] | 10 [5-21] | 9 [3-20] | 7 [1-16] |
| **Outcome** | | | | | | | |
| 30-day mortality | 2446 (31) | 701 (21) | 486 (29) | 518 (38) | 347 (45) | 287 (55) | 107 (64) |
| 90-day mortality | 3021 (39) | 926 (28) | 629 (37) | 630 (46) | 400 (52) | 324 (62) | 112 (67) |

Data are expressed as n (%) or median [IQR]. [a] Based on international classification of diseases codes only. [b] According to Danish regulation, frequencies smaller than five patients not being zero and the corresponding total number of patients are not reported. [c] Data missing for 6,088 (77%) patients. [d] Data missing for 383 (5%) patients. [e] Data missing for 5094 (63%) patients. [f] Data missing for 171 (2%) patients. [g] Data missing for 445 (6%) patients.

**Additional file 1 -** Prognostic impact of elevated lactate levels on mortality in critically ill patients with and without preadmission metformin treatment: a Danish registry-based cohort study

**Table S15 – Thirty-day mortality, hazard ratios, and the relative excess risk due to interaction by lactate category for metformin nonusers and users, when comparing two subgroups based on preadmission estimated glomerular filtration rate**

| **eGFR ≥60 ml/min/1.72m²** | **Events / no. at risk** | | **Crude HR (95% CI)** | | **Adjusted HR (95% CI)[a]** | | **RERI (95% CI)[b]** |
|---|---|---|---|---|---|---|---|
| Lactate (mmol/L) | Metformin nonusers | Metformin users | Metformin nonusers | Metformin users | Metformin nonusers | Metformin users | |
| <1.4 | 928 / 8297 (11%) | 74 / 732 (10%) | Reference | 0.89 (0.70 to 1.13) | Reference | 0.85 (0.67 to 1.08) | |
| 1.4 to 2.0 | 750 / 4842 (15%) | 70 / 557 (13%) | 1.42 (1.29 to 1.56) | 1.13 (0.89 to 1.44) | 1.45 (1.32 to 1.60) | 1.11 (0.87 to 1.43) | -0.19 (-0.54 to 0.16) |
| 2.0 to 3.2 | 748 / 3643 (21%) | 63 / 398 (16%) | 1.94 (1.76 to 2.13) | 1.45 (1.12 to 1.87) | 1.99 (1.81 to 2.19) | 1.44 (1.10 to 1.87) | -0.40 (-0.84 to 0.04) |
| 3.2 to 5.0 | 600 / 1986 (30%) | 57 / 248 (23%) | 3.06 (2.76 to 3.39) | 2.17 (1.66 to 2.83) | 3.20 (2.89 to 3.55) | 2.03 (1.54 to 2.68) | -1.01 (-1.65 to -0.38) |
| 5.0 to 10.0 | 534 / 1295 (41%) | 68 / 211 (32%) | 4.67 (4.20 to 5.19) | 3.38 (2.64 to 4.32) | 4.89 (4.39 to 5.44) | 3.24 (2.52 to 4.17) | -1.50 (-2.41 to -0.58) |
| ≥10.0 | 285 / 476 (60%) | 45 / 85 (53%) | 8.68 (7.60 to 9.91) | 6.86 (5.08 to 9.25) | 9.93 (8.68 to 11.37) | 7.04 (5.18 to 9.57) | -2.74 (-5.17 to -0.31) |
| **eGFR <60 ml/min/1.72m²** | **Events / no. at risk** | | **Crude HR (95% CI)** | | **Adjusted HR (95% CI)[a]** | | **RERI (95% CI)[b]** |
| Lactate (mmol/L) | Metformin nonusers | Metformin users | Metformin nonusers | Metformin users | Metformin nonusers | Metformin users | |
| <1.4 | 649 / 3029 (21%) | 52 / 277 (19%) | Reference | 0.87 (0.66 to 1.15) | Reference | 1.01 (0.76 to 1.35) | |
| 1.4 to 2.0 | 446 / 1511 (30%) | 40 / 177 (23%) | 1.46 (1.29 to 1.64) | 1.06 (0.77 to 1.45) | 1.47 (1.30 to 1.66) | 1.18 (0.85 to 1.63) | -0.30 (-0.79 to 0.19) |
| 2.0 to 3.2 | 474 / 1196 (40%) | 44 / 159 (28%) | 2.14 (1.90 to 2.41) | 1.37 (1.01 to 1.86) | 2.14 (1.90 to 2.41) | 1.60 (1.17 to 2.18) | -0.56 (-1.15 to 0.04) |
| 3.2 to 5.0 | 322 / 694 (46%) | 25 / 82 (30%) | 2.66 (2.32 to 3.04) | 1.52 (1.02 to 2.27) | 2.70 (2.36 to 3.09) | 1.72 (1.15 to 2.58) | -0.99 (-1.79 to -0.19) |
| 5.0 to 10.0 | 258 / 447 (58%) | 29 / 76 (38%) | 3.97 (3.43 to 4.58) | 2.10 (1.45 to 3.05) | 4.28 (3.70 to 4.95) | 2.58 (1.77 to 3.77) | -1.71 (-2.85 to -0.58) |
| ≥10.0 | 94 / 130 (72%) | 13 / 38 (34%) | 6.64 (5.35 to 8.25) | 1.93 (1.11 to 3.34) | 6.95 (5.59 to 8.65) | 2.67 (1.53 to 4.64) | -4.29 (-6.39 to -2.20) |

Hazard ratio (HR) with 95% confidence interval (95% CI) was computed using Cox proportional regression analysis based on a multiple imputed dataset including patients with a preadmission estimated glomerular filtration rate (eGFR) ≥60 ml/min/1.72m² or <60 ml/min/1.72m².

[a] Adjusted for age, gender, last HbA1c measurement within 4 weeks before ICU admission, and previous diagnosis of myocardial infarction, congestive heart failure, peripheral artery disease, cerebrovascular disease, dementia, connective tissue disease, peptic ulcer disease, chronic pulmonary disease, mild to severe

liver disease, any tumor, metastatic solid tumor, leukemia, and lymphoma, respectively, 10 years before ICU admission. Codes used for each variable are stated in Appendix 1.

[b] Relative excess risk due to interaction (RERI) quantifies interaction on an additive scale, which approaches zero in the absence of interaction.

**Additional file 1 -** Prognostic impact of elevated lactate levels on mortality in critically ill patients with and without preadmission metformin treatment: a Danish registry-based cohort study

**Appendix 1 – List of hospitals included in the study**

| | Number of ICU admissions in 2017/2018* |
|---|---|
| **Denmark** | 29,748 |
| **Northern Denmark Region** | **3200 (11%)** |
| Aalborg Universitetshospital | 2456 |
| Regionshospital Nordjylland, Thisted | 206 |
| Regionshospital Nordjylland, Hjørring | 538 |
| **Central Denmark Region** | **7776 (26%)** |
| Aarhus Universitetshospital | 3558 |
| Regionshospitalet Viborg | 1238 |
| Regionshospitalet Randers | 873 |
| Regionshospitalet Herning | 738 |
| Regionshospitalet Horsens | 664 |
| Regionshospitalet Holstebro | 511 |
| Regionshospitalet Silkeborg | 194 |

Data are provided as frequencies and are based on the national annual rapport 2017/2018 of the Danish Intensive Care Database, which can be obtained from https://www.sundhed.dk/content/cms/12/4712_did_aarsrapport_2018.pdf
* number of ICU admissions are provided to give an example of the size of the population that was studied, and does not provide any details whether lactate was measured or whether these patients were included in the cohort.

**Additional file 1 -** Prognostic impact of elevated lactate levels on mortality in critically ill patients with and without preadmission metformin treatment: a Danish registry-based cohort study

**Appendix 2 – Codes used in this study**

| **Characteristic** | **Code** |
|---|---|
| **Preadmission morbidity, based on diagnosis codes according to the International Classification of Diseases, 10th revision (ICD-10).** | |
| Myocardial infarction | I21-I23 |
| Congestive heart failure | I11.0, I13.0, I13.2, I50 |
| Peripheral vascular disease | I70-I74, I77 |
| Cerebrovascular disease | I60-I69, G45, G46 |
| Dementia | F00−F03, F05.1, G30 |
| Chronic pulmonary disease | J40-67, J68.5, J70.1, J84.1, J92.0, J96.1, J98.2, J98.3 |
| Connective tissue disease | M05, M06, M08, M09, M30-M36, M45, D86 |
| Ulcer disease | K22.1, K25-K28 |
| Mild liver disease | B18, K70.0-K70.3, K70.9, K71, K73, K74, K76.0 |
| Moderate to severe liver disease | B15.0, B16.0, B16.2, B19.0, K70.4, K72, K76.6, I85 |
| Diabetes without end-organ failure | E10.0, E10.1, E10.9, E11.0, E11.1, E11.9 |
| Diabetes with end-organ failure | E10.2-E10.8, E11.2-E11.8, |
| Diabetes | E10-13, G63.2, H36.0, N0.83, O24 (except O24.4), G63.2, H36.0, N08.3 |
| Moderate to severe renal disease | I12, I13, N00−N05, N07, N11, N14, N18−N19, Q61 |
| Hemiplegia | G81, G82 |
| Any tumor | C00-C96 (except C44) |
| Metastatic solid tumor | C76−C80 |
| Leukemia | C91-C95 |
| Lymphoma | C81-C85, C88, C90, C96 |
| AIDS | B21-B24 |
| PCOS | E28.2 |
| **Primary diagnosis during current hospitalisation (diagnostic category).**<br>**Diagnosis codes are according to the International Classification of Diseases, 10th revision (ICD-10).** | |
| Cardiovascular | I03-I29, I30.0, I30.8, I30.9, I31, I34-I90 |
| Respiratory, incl. pneumonia | J00−J99 (except J36, J39.0, J86), A48.1, A70.9 |
| Infection or sepsis, excl. pneumonia | A00−B99 (without A48.1, A70.9), B37.7, G00−G07, I00−I02, I30.1, I32.0, I33, I38, I40.0, J00−J06, J36, J39.0, J10−J11, J20−J22, J85.1, J86, K35, K37, K57.0, K57.2, K57.4, K57.8, K61, K63.0, K65.0, K65.9, K67, K75.0, K75.1, K80.0, K80.3, K80.4, K81.0, K81.9, K83.0, L00−L03, L05−L08, M00, M01, M86, N10, N12, N15.1, N30, N39.0, N41, N45, N70−N77 |
| Gastrointestinal and liver disease | K00−K99 without K35, K37, K57.0, K57.2, K57.4, K57.8, K61, K63.0, K65.0, K65.9, K67, K75.0, K75.1, K80.0, K80.3, K80.4, K81.0, K81.9, K83.0 |
| Neoplasms | C00−D89 |
| Trauma and poisoning | S00−T98 |
| Endocrinology | E00-E90, O24 (except O24.4), G63.2, H36.0, N08.3 |
| Other | all codes not included in other categories |
| Lactic acidosis | E87.2A |

**Additional file 1 -** Prognostic impact of elevated lactate levels on mortality in critically ill patients with and without preadmission metformin treatment: a Danish registry-based cohort study

| | |
|---|---|
| **Preadmission drug use according to the Anatomical Therapeutic Classification** | |
| Metformin | A10BA02, A10BD02, A10BD03, A10BD05, A10BD07, A10BD08, A10BD10, A10BD11, A10BD13-A10BD18, A10BD20, A10BD22 |
| Sulfonylurea | A10BB, A10BC, A10BD04, A10BD06 |
| Insulin | A10A |
| Other antihyperglycemic drugs | A10B, except for the codes stated above |
| **Danish Health Care Classification System procedure codes** | |
| Intensive care therapy or observation | NABB / NABE |
| Mechanical ventilation | BGDA0 |
| Use of inotropes of vasopressors | BFHC92 A-E, BFHC93 A-C, BFHC95 |
| Initiation of renal replacement therapy | BJFD0 |
| Chronic dialysis | BJFD2 |
| Simplified Acute Physiology Score II | ZRRB |
| **Blood tests according to Nomenclature for Properties and Units codes and local analysis codes** | |
| Lactate | NPU02544, NPU03942-NPU03944, NPU17374, NPU18325, NPU22097, NPU22098, ASS00121<br><br>Northern Denmark Region<br>ASS00113, ASS00120<br><br>Central Denmark Region<br>AAB00225, ASS00930<br><br>Analysis number: 3943, 3944, 110255, 113943, 1511609, 1813944, |
| Glucose | NPU02187, NPU02192, NPU02193, NPU02195, NPU04093, NPU08509, NPU08520-NPU08567, NPU08869- NPU08916, NPU08972, NPU10072, NPU10074, NPU10076, NPU10077, NPU10082, NPU21531, NPU21533, NPU22069-NPU22089, NPU22095, NPU22099-NPU22126,<br>DNK35842<br><br>Northern Denmark Region<br>ASS00203, ASS00204, ASS00251, ASS00254<br><br>Analysis number: 1411613, 1411614, 1411616, 1411618, 1411621, 1411622<br><br>Central Denmark Region<br>AAA00061-AAA00063, AAA00065, AAA00067, AAA00163, AAA00209, AAA00303, AAA00308, AAA00311, AAA00314, AAA00317, AAA00320, AAA00321, AAA00323, AAA00378, AAA00379, AAA00400, AAB00226, SIL93034<br><br>Analysis number: 127, 714, 2187, 2192, 2195, 8544-8567, 8904, 8909, 8914, 8916, 8972, 10065, 10066, 10070, 10501-10503, 14517, 19720, 21531, 22069, 27294, 35842, 110624, 110840, 111624, 113016, 114301-114308, 114325, 116880, 122432, 1510941, 1511500-1511549, 1511591, 1523098, 1610501-1610503, 1810302, 1810308, 1810309, 1810311, 1810312, 1810316, 1810322, 1812187, 1812195, 1817017, 1817018, 1817349. |
| Creatinine | NPU18016, NPU01807, NPU04998, ASS00354, ASS00355, ASS00356<br><br>Central Denmark Region: |

| | |
|---|---|
| | AAB00327<br><br>Analysis code: 716, 1807, 4998, 5224, 18016, 110266, 1511235, 1610154, 1611807, 1710301, 1711807, 1811807, 1817156 |
| HbA1c | NPU27300, NPU02307, NPU03835, NPU27300, DNK35249<br><br>Northern Denmark Region<br>Analysis number: 1312155, 1412155<br><br>Central Denmark Region<br>AAA00740, AAB00061, AAB00091, AAB00092, EQA00330-EQA00332, EQA00335, RIN00164, VEA00104<br><br>Analysis number: 153, 154, 159, 160,  2499, 3855, 113835, 1510994, 1610135, 1712307, 1812307, 1512155 |

**Additional file 1 -** Prognostic impact of elevated lactate levels on mortality in critically ill patients with and without preadmission metformin treatment: a Danish registry-based cohort study

**Appendix 3 – Covariates entered in the models**

| **Cohort** | **Cox regression model** | **Spline Cox regression model** |
|---|---|---|
| Full cohort | Survival ~ metformin/lactate category + age + male sex + preadmission mean creatinine + HbA1c + preadmission myocardial infarction + preadmission congestive heart failure + preadmission peripheral artery disease + preadmission cerebrovascular disease + preadmission dementia + preadmission connective tissue disease + preadmission peptic ulcer disease + preadmission chronic pulmonary disease + preadmission liver disease (mild to severe) + preadmission any tumor + preadmission metastatic solid tumor + preadmission leukemia + preadmission lymphoma | Survival ~ rcs(lactate, 4) + metformin use + rcs(age,4) + male sex + rcs(preadmission mean creatinine,4) + rcs(HbA1c, 4) + preadmission myocardial infarction + preadmission congestive heart failure + preadmission peripheral artery disease + preadmission cerebrovascular disease + preadmission dementia + preadmission connective tissue disease + preadmission peptic ulcer disease + preadmission chronic pulmonary disease + preadmission liver disease (mild to severe) + preadmission any tumor + preadmission metastatic solid tumor + preadmission leukemia + preadmission lymphoma |
| Diabetes mellitus | Survival ~ metformin/lactate category + age + male sex + preadmission mean creatinine + HbA1c + preadmission myocardial infarction + preadmission congestive heart failure + preadmission peripheral artery disease + preadmission cerebrovascular disease + preadmission dementia + preadmission connective tissue disease + preadmission peptic ulcer disease + preadmission chronic pulmonary disease + preadmission liver disease (mild to severe) + preadmission any tumor + preadmission metastatic solid tumor + preadmission leukemia + preadmission lymphoma + sulfonylurea use + insulin use + other antihyperglycemic drug use | Survival ~ rcs(lactate, 4) + metformin use + rcs(age,4) + male sex + rcs(preadmission mean creatinine,4) + rcs(HbA1c, 4) + preadmission myocardial infarction + preadmission congestive heart failure + preadmission peripheral artery disease + preadmission cerebrovascular disease + preadmission dementia + preadmission connective tissue disease + preadmission peptic ulcer disease + preadmission chronic pulmonary disease + preadmission liver disease (mild to severe) + preadmission any tumor + preadmission metastatic solid tumor + preadmission leukemia + preadmission lymphoma + sulfonylurea use + insulin use + other antihyperglycemic drug use |
| Elective surgery | Survival ~ metformin/lactate category + age + male sex + preadmission mean creatinine + HbA1c + Charlson comorbidity index category | Survival ~ rcs(lactate, 4) + metformin use + rcs(age,4) + male sex + rcs(preadmission mean creatinine,4) + rcs(HbA1c, 4) + Charlson comorbidity index category |
| Preadmission renal function | Survival ~ metformin/lactate category + age + male sex + HbA1c + preadmission myocardial infarction + preadmission congestive heart failure + preadmission peripheral artery disease + preadmission cerebrovascular disease + preadmission dementia + preadmission connective tissue disease + preadmission peptic ulcer disease + preadmission chronic pulmonary disease + preadmission liver disease (mild to severe) + preadmission any tumor + preadmission metastatic solid tumor + preadmission leukemia + preadmission lymphoma | Survival ~ rcs(lactate, 4) + metformin use + rcs(age,4) + male sex + rcs(HbA1c, 4) + preadmission myocardial infarction + preadmission congestive heart failure + preadmission peripheral artery disease + preadmission cerebrovascular disease + preadmission dementia + preadmission connective tissue disease + preadmission peptic ulcer disease + preadmission chronic pulmonary disease + preadmission liver disease (mild to severe) + preadmission any tumor + preadmission metastatic solid tumor + preadmission leukemia + preadmission lymphoma |

**Additional file 1 -** Prognostic impact of elevated lactate levels on mortality in critically ill patients with and without preadmission metformin treatment: a Danish registry-based cohort study

### Appendix 4 – Additional methods regarding subgroups

The model used in the cohort including patients with diabetes mellitus cumulatively included different antihyperglycemic treatments as covariables. Most elective surgical patients did not have elevated lactate levels. Therefore, we categorised the lactate level according to the reference value (<2 and ≥2 mmol/L). To ensure an adequate sample size regarding the number of outcome events relative to parameters used in the model of elective surgery patients, the Charlson Comorbidity Index instead of individual preadmission comorbidities was entered as covariable. The model to assess the association of lactate with mortality in patients stratified according to preadmission renal function was the same as the model used in our primary analysis, except the model was not adjusted for preadmission renal function.
